# Transcriptomic analysis unveils bona fide molecular signatures of microglia under conditions of homeostasis and viral encephalitis

**DOI:** 10.1186/s12974-024-03197-2

**Published:** 2024-08-17

**Authors:** Felix Mulenge, Olivia Luise Gern, Lena Mareike Busker, Angela Aringo, Luca Ghita, Inken Waltl, Andreas Pavlou, Ulrich Kalinke

**Affiliations:** 1 Institute for Experimental Infection Research, Centre for Experimental and Clinical Infection Research, a joint venture between The Helmholtz-Centre for Infection Research, Hannover Medical School, TWINCORE, Feodor-Lynen-Str. 7, 30625 Hannover, Germany; 2 Department of Pathology, University of Veterinary Medicine Hannover, 30559 Foundation, Hannover, Germany; 3 Genentech, South San Francisco, CA 94080 USA; 4 Cluster of Excellence RESIST (EXC 2155), Hannover Medical School, 30625 Hannover, Germany

## Abstract

Microglia serve as a front-line defense against neuroinvasive viral infection, however, determination of their actual transcriptional profiles under conditions of health and disease is challenging. Here, we used various experimental approaches to delineate the transcriptional landscape of microglia during viral infection. Intriguingly, multiple activation genes were found to be artificially induced in sorted microglia and we demonstrated that shear stress encountered during cell sorting was one of the key inducers. Post-hoc analysis revealed that publicly available large-scale single-cell RNA sequencing datasets were significantly tainted by aberrant signatures that are associated with cell sorting. By exploiting the ribosomal tagging approach, we developed a strategy to enrich microglia-specific transcripts by comparing immunoprecipitated RNA with total RNA. Such enriched transcripts were instrumental in defining *bona fide* signatures of microglia under conditions of health and virus infection. These unified microglial signatures may serve as a benchmark to retrospectively assess ex vivo artefacts from available atlases. Leveraging the microglial translatome, we found enrichment of genes implicated in T-cell activation and cytokine production during the course of VSV infection. These data linked microglia with T-cell re-stimulation and further underscored that microglia are involved in shaping antiviral T-cell responses in the brain. Collectively, our study defines the transcriptional landscape of microglia under steady state and during viral encephalitis and highlights cellular interactions between microglia and T cells that contribute to the control of virus dissemination.

**Supplementary Information:**

The online version contains supplementary material available at 10.1186/s12974-024-03197-2.

## Introduction

Microglia are resident immune cells of the central nervous system (CNS) that arise from erythromyeloid progenitors, which seed the brain parenchyma during embryonic development [1]. They are highly plastic in nature allowing them to continually survey the CNS milieu and to establish brain homeostasis by regulating synaptic wiring, myelination, vasculogenesis, and remodeling neuronal circuits [2]. Moreover, microglia are central mediators that respond subtly and sometimes grossly to various neuropathologies such as injury, inflammation, cancer and neurodegeneration [3–6], by instrumentalization of signaling mechanisms that are not fully resolved, yet. To better understand the diverse multifunctional roles of microglia, it is critical to precisely illuminate transcriptomic signatures of certain microglial phenotypes. With the recent advent of novel sequencing technologies, cell isolation approaches, and computational frameworks, a significant expansion of high-throughput profiling of different cell types was promoted, which provided new insights into the biology of cells within their tissue context [7, 8]. Furthermore, these large-scale transcriptomic datasets serve as a reference atlas providing a high-level overview of expression patterns of cell subsets that would otherwise be too costly or time-consuming for individual laboratories to generate. These data are, however, not without caveats.

A critical step in transcriptomic studies is the isolation and preparation of pure suspensions of the relevant cell subset. This is particularly challenging for microglia, since their low abundance in the brain often results in little cell yields. Microglia can be enriched from brain cell preparations by fluorescence-activated cell sorting (FACS) [9]. Combined immunolabeling of core markers such as CD11b^+^ and CD45^low^ allows discrimination of microglia from recirculating monocytes [4]. However, microglial CD45 levels may change after brain injury and during brain disease [10], thus making discrimination of microglia from monocytes difficult [11]. Substantial efforts have been undertaken to generate inducible mouse lines expressing fluorescent markers under the control of microglia-specific promoters such as CX3CR1, HEXB, TMEM119, and P2RY12 [12–15]﻿. These models allow sorting of microglia without the need to immunolabel the cells by antibodies. The ribosomal tagging approach (RiboTag) is an alternative way to studying microglia-specific gene expression profiles from complex tissue. This method relies on Cre recombinase mediated expression of a haemagglutinin (HA) tag fused to the core ribosomal protein 22 (Rpl22) [16], allowing the pull-down of mRNA bound to HA-tagged ribosomes and determining the translational profile of brain resident myeloid cells [17]. In addition, various microglia in vitro models, including primary cultivated cell cultures, stem cell-derived microglia cultures, organoid, and immortalized microglia cell lines have been developed [18–21]. Such in vitro systems are assumed to model microglia physiology and function in an experimentally amenable manner.

A key pursuit in molecular neuroscience is to gain a mechanistic understanding of orchestrated microglial responses in health and disease. However, the heterogeneity of microglial signatures, as detected in various studies, the discrepancies between transcriptomic datasets and the lack of harmonized microglia isolation protocols have made it difficult to achieve a consensus on the main features, pathways and effects of microglia responses to pathological insults. Here, we performed a side-to-side comparative analysis of the transcriptomes obtained from in vitro cultivated microglia, sorted microglia, and the microglial translatome determined by the RiboTag approach, to discern specific molecular triggers and signatures that are defining microglia under homeostatic and inflammatory conditions. Our data illustrate that conventional bulk RNA transcriptomes of sorted microglia contain ex vivo aberrant activation signatures. Such “spurious” signatures were also detected in post-hoc analysis of publicly available large-scale single-cell RNA sequencing (scRNA-seq) datasets or atlases, underscoring the need to develop approaches that identify, compensate and/or avoid such biases. In brief, we incorporated the concept of enrichment of relevant gene signatures by comparing immunoprecipitated RNA with total RNA in the RiboTag approach and assessing the specificity of given transcripts known to be abundantly present in the tissue. Transcripts that are enriched are more likely specifically expressed within microglia, whereas transcripts that are depleted are more likely to be expressed in other cell types (i.e., non-myeloid) contained within the tissue. This approach allowed generation of accurate in situ transcriptional profiles of microglia under homeostatic conditions and after vesicular stomatitis virus (VSV) infection that will be especially useful in retrospective removal of ex vivo artefacts present in existing dataset. Leveraging the microglial translatome, we demonstrated that upon VSV infection, microglia adopt unique expression profiles implicated in cytokine production and antigen presentation. We further reported dramatic influx of monocytes, neutrophils, B cells, natural killer (NK) cells, and T cells in the CNS of VSV-infected animals compared to controls, demonstrating substantial shift in brain immune cell composition. Indeed, we found that infiltrated T cells surrounded the dense conglomerates of microglia, suggesting that T cells intimately interact with activated microglia. Together, this study investigates protective mechanisms orchestrated by microglia in severe neuroinflammation and CNS infection.

## Results

### Adoption of different experimental approaches variably impacts microglia gene expression profiles

**Fig. 1:**
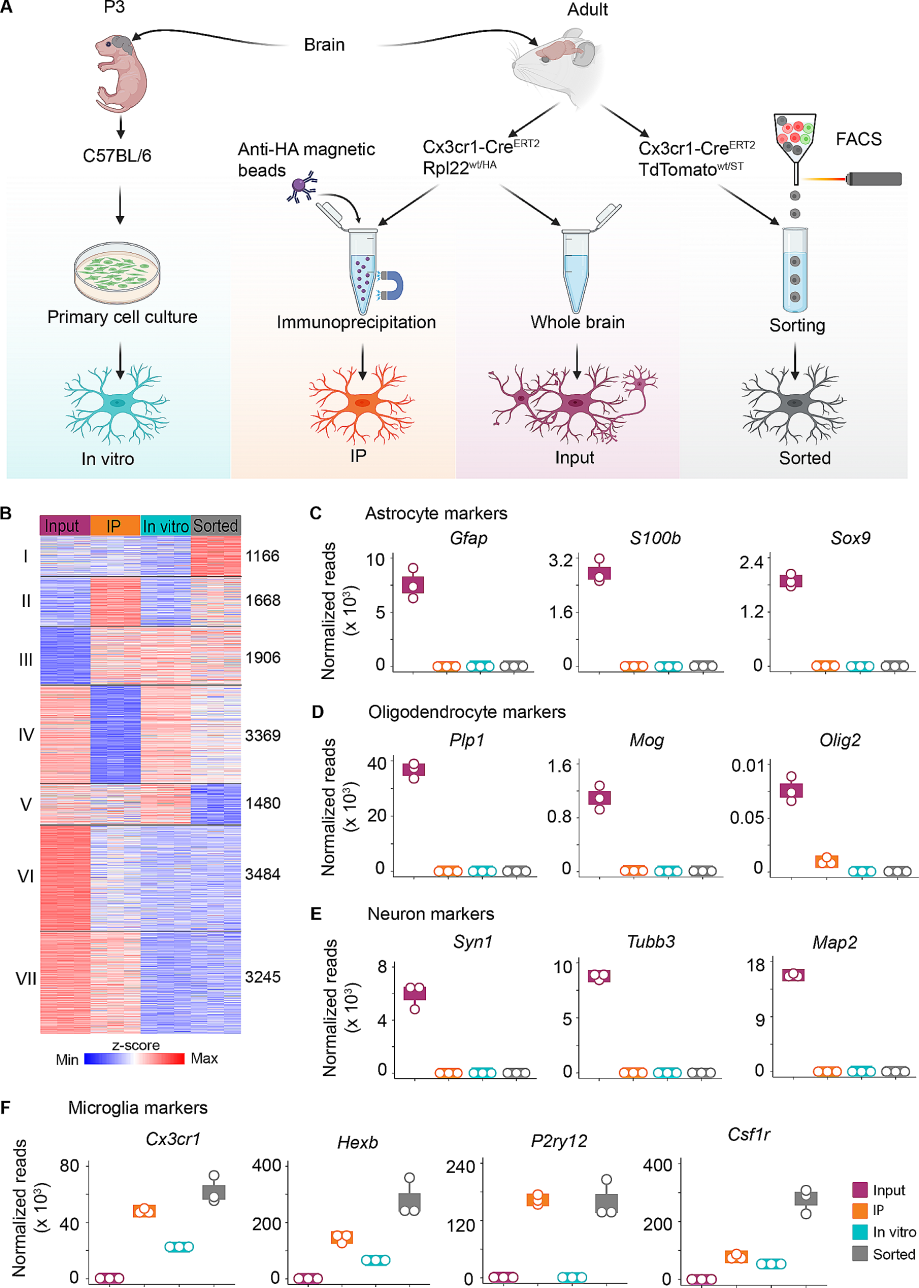
Comparative analysis of transcriptomes and translatomes reveals diverse cellular profiles of microglia. **A**, Schematic depiction of the experimental models that were used to study the transcriptome and translatome of microglia. In vitro: Mixed glia cultures were prepared from P3 pups and microglia harvested after 10 days. IP: The brain of tamoxifen treated CX3CR1-Cre^ERT2+/−^Rpl22^wt/HA^ RiboTag mice was extracted, microglia-specific HA-tagged ribosomes were pulled down, and the attached RNA was isolated. Input: RNA from whole brain lysate was isolated. Sorted: Immune cells from the brain of tamoxifen-treated CX3CR1-Cre^ERT2+/−^TdTomato^wt/ST^ mice were isolated and TdTomato-positive microglia were sorted. **B**, Heatmap representing k-means clustering of highly enriched and de-enriched genes in RNA-seq analysis of input, IP, in vitro, and sorted microglia. Red represents enriched and blue de-enriched gene expression levels. Boxplots with overlaid dotplots of normalized gene counts for **(C)** astrocyte, **(D)** oligodendrocyte, **(E)** neuron, and **(F)** microglia lineage markers. In each group three individual mice or triplicates of microglia cultures were analyzed (*n* = 3). Each boxplot represents interquartile range, while dotplots illustrate individual data points. Normalized reads represent read counts that have been normalized by DESeq2 (for details see Materials and Methods)

Microglia are brain resident myeloid cells that can adopt a wide range of different functions in health and disease. Here, we aimed at determining the *bona fide* molecular signatures of microglia under homeostatic conditions and following VSV infection. To address this, we studied the transcriptomes of microglia isolated from different experimental models and performed a combinatorial analysis of the retrieved data. First, we cultivated mixed glial cultures from brain cortices of P3 mouse pups and harvested in vitro microglia after 10 days (hereafter denoted “*in vitro”)* (Fig. 1A). Secondly, we exploited the RiboTag approach, i.e., we isolated the brain from CX3CR1-Cre^ERT2+/−^Rpl22^wt/HA^ mice 8 weeks after tamoxifen treatment, prepared tissue lysates, and directly immunoprecipitated ribosome-bound RNA from brain-resident myeloid cells (hereafter denoted “immunoprecipitated [IP]”), or we isolated the total RNA from brain homogenates (hereafter denoted “input”). Lastly, we included RNA-seq data from our lab [4], which were retrieved from isolated brain resident immune cells of tamoxifen treated CX3CR1-Cre^ERT2+/−^TdTomato^wt/ST^ mice and sorted TdTomato positive myeloid cells (hereafter denoted “sorted”). After RNA extraction from material obtained by the different approaches, RNA-seq was performed. Initially, we analyzed microglia from mock-treated samples to decipher baseline transcriptional profiles of homeostatic microglia. As sample processing and sequencing was performed at different times, we first used ComBat-seq [22] to model batch effects and other latent sources of noise. After batch effect adjustment, we performed differential analysis (log2-fold change >|1|, padj < 0.05) by employing the likelihood ratio test (LRT) and the resulting genes were subjected into k-means clustering. This analysis revealed the presence of 7 distinct expression patterns amongst input, IP, in vitro, and sorted samples (Fig. 1B). Cluster I comprised genes that were enriched in the transcriptome of sorted microglia, whereas cluster II consisted of 1,668 genes that were enriched in IP and sorted microglia and therefore can be annotated as CX3CR1-dependent. Cluster III comprised 1,906 genes that were highly abundant in IP, in vitro, and sorted samples, but that were less abundant in input samples, and therefore these genes were identified as being microglia-associated. Cluster IV consisted of genes that were prominently enriched in the input, in vitro, and sorted samples. In cluster V input, IP, and in vitro samples displayed 1,480 prominent genes that were absent in sorted microglia. Cluster VI comprised 3,484 genes that were abundantly expressed in input samples and corresponded to genes that were typically expressed in non-myeloid cells of the CNS. In cluster VII, 3245 genes were enriched in input and IP samples, and de-enriched in in vitro and sorted microglia, respectively.

To assess the cell type-specificity of the obtained translatomes, we evaluated the expression of well-known neuronal and glia-specific marker genes. Quantification of normalized gene counts revealed that the astrocytic marker genes *Gfap*,* S100b*, and *Sox9*, the oligodendrocytic marker genes *Plp1*,* Mog*, and *Olig2*, and the neuronal marker genes *Syn1*, *Tubb3*, and *Map2* were highly expressed in input samples (Fig. 1C-E). In contrast, the core microglia marker genes *Cx3cr1*, *Hexb*, *P2ry12*, and *Csf1r* were abundantly expressed in IP, in vitro, and sorted microglia (Fig. 1F), illustrating that all three methods were suitable to enrich RNA from microglia and that they are amendable to investigate microglia RNA expression profiles.

### Transcriptomic and translatomic analyses of microglia reveal confounding gene signatures that are associated with cell sorting

**Fig. 2:**
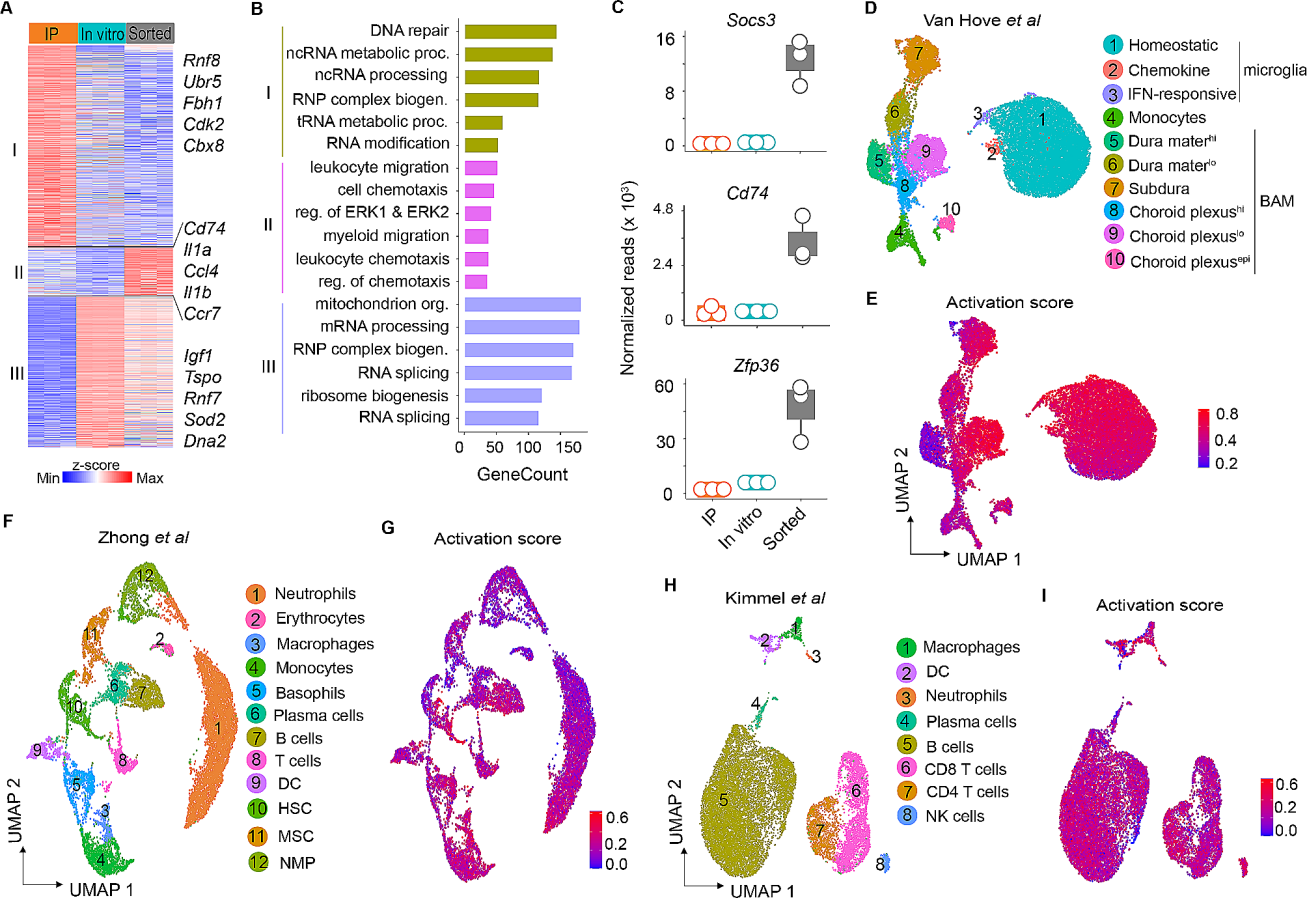
Sorted microglia exhibit artificially induced transcriptional alterations. **A**, Heatmap representing k-means re-clustering of IP, in vitro, and sorted microglia, showing cluster-specific gene enrichment and de-enrichment. **B**, Gene ontology analysis displaying top five pathways associated with genes retrieved from clusters in A. **C**, Boxplots with dotplot overlays representing normalized gene counts of selected genes from cluster II, revealing high expression of activation-related genes in sorted samples (for additional details see caption of Fig. 1). **D**, UMAP plot of scRNA-seq data [23] from 16,506 cells that were analyzed from whole brain, depicted are myeloid cell subsets. **E**, UCell score distribution for cell activation signatures retrieved from bulk RNA-seq in cluster II shown in UMAP space. **F**, UMAP representation of scRNA-seq analysis of 11,850 cells from bone marrow highlighting major cell types. **G**, UCell score distribution in UMAP space for cell activation signatures in bone marrow cells. **H**, UMAP embedding of scRNA-seq data from 14,146 cells analyzed from spleen, showing key immune cell types. **I**, UMAP plot showing UCell score distribution of cell activation signatures in splenocytes. DC, dendritic cells; HSC, hematopoietic stem cells; MSC, mesenchymal stem cells; NMP, neutrophil-myeloid progenitor cells; BAMs, border associated macrophages; IFN, interferon

To address whether the similarities and differences in gene signatures observed in the applied experimental models reflected true biological effects, we performed unbiased k-means clustering of differentially regulated genes in IP, in vitro, and sorted microglia samples. Three clusters were detected (Fig. 2A), while cluster I is defined by genes that were highly expressed in IP microglia relative to in vitro and sorted microglia, cluster II comprised genes that were enriched in sorted samples including genes encoding for chemokines (*Ccr7*,* Ccl4*) and interleukins (*Il1a*,* Il1b*). Cluster III comprised genes that were similarly enriched in in vitro and sorted samples. Microglial cluster-specific genes in cluster I and cluster III highlight differences between transcriptomes and translatomes. Using Gene Ontology (GO) terms, we identified cluster I and III-specific genes that are implicated in DNA repair, ncRNA processing, mRNA processing, and RNA splicing functions (Fig. 2B). Importantly, genes involved in leukocyte migration, cell chemotaxis and myeloid leukocyte migration were the most highly overrepresented in cluster II. When we probed for specific genes associated with the aforementioned pathways, we detected abundant expression of stress-induced genes (*Zfp36*, *Dusp1*,* Jun*) as well as immune-related genes (*Socs3*,* Cd74*,* Ccl4*,* Nfkbiz*,* Ccl3*) in sorted microglia and to a lesser extent in IP and in vitro samples (Fig. 2C and Fig. S1A), suggesting a transcriptionally activated state of the sorted microglia.

To analyze whether microglial activation signatures were also present in large-scale transcriptomics studies, we first performed a re-analysis of scRNA-seq data of brain resident immune cells published by Van Hove et al. [23]. Unsupervised clustering identified 14 transcriptionally distinct clusters representing all immune cell types known to be present in the CNS (Fig. S1B). Sub-clustering of microglia, border associated macrophages (BAMs) and monocytes revealed 10 discrete myeloid cell subpopulations (Fig. 2D), including three microglia clusters that correspond to homeostatic microglia (*Hexb*,* P2ry12*,* Tmem119*), chemokine microglia (*Il1a*,* Tnf*,* Ccl3*) and IFN-responsive microglia (*Ifit3*,* Isg15*,* Ccl12*) (Fig. S1C). The latter represent surveying cells that can rapidly respond to any brain dysfunction, infection or damage [24]. To visualize the cell types in the dataset that show enrichment of cell activation signatures, we performed gene module scoring by using UCell [25]. We generated “activation-induced signatures” by applying the consensus of genes that was retrieved from cluster II in Fig. 2A as well as previously published data [17, 26–28] (Supplemental Table 1). The enrichment analysis projected onto UMAP demonstrated that activation-induced signatures were mostly enriched in myeloid cell subpopulations, albeit to different extents (Fig. 2E). Moreover, marker expression analysis revealed that the identified cell activation genes *Jun*, *Socs3*, *Nfkbia*, *Ccrl2*, *Dusp1*, and *Zfp36* were highly expressed in microglia and BAMs and low in monocytes (Fig. S1D).

We next asked whether these spurious signals were unique to CNS-resident myeloid cells, or whether they were also detected in peripheral immune cells. To this end, we analyzed a recently published scRNA-seq immune cell map that included T and B cells, NK cells, and neutrophils from murine bone marrow [29] and spleen samples [30]. Clustering of these datasets reproduced cell type labels as described in the original publications (Fig. 2F and H). Unbiased gene module scoring of activation-induced signatures on all cell types indicated that nearly all hematopoietic cells in the bone marrow (Fig. 2G and Fig. S2D) as well as splenocytes (Fig. 2I and Fig. S2E) were enriched for cells carrying activation signatures. Thus, aberrant transcriptional signatures are prevalent in immune cells from the CNS and the periphery. Moreover, these spurious signals appeared to be systemically present not only in study-specific datasets but also in reference atlases that are intended to be used as reference-based methods for cell type annotation.

### Microglia show induction of transcriptional activation following flow cytometric sorting

To pinpoint the causative sources of artefacts in microglia transcriptomic profiles, we next leveraged publicly available scRNA-seq datasets of microglia isolated by different protocols with single variations in key experimental steps and performed in-silico integration. These datasets encompassed samples from (i) Van Hove et al. [23] who applied standard enzymatic tissue dissociation and flow cytometric sorting, (ii) Tepe et al. [26] who applied standard enzymatic tissue dissociation, (iii) Hammond et al. [31] who applied mechanical tissue dissociation and flow cytometric sorting under ice-cold conditions, and (iv) Marsh et al. [27] who used standard enzymatic tissue dissociation, flow cytometric sorting, and included transcriptional inhibitors. Datasets from Van Hove et al., Hammond et al., and Marsh et al. are based on CD45^+^ cells that were sorted from the mouse brain (Fig. S2A and S2B), while Tepe et al. included all CNS resident cells in their analysis (Fig. S2C). The integration pipeline started by separately processing each dataset and annotating CNS cell types in line with the source study, and subsequently selecting the immune cell clusters that represent microglia.

**Fig. 3:**
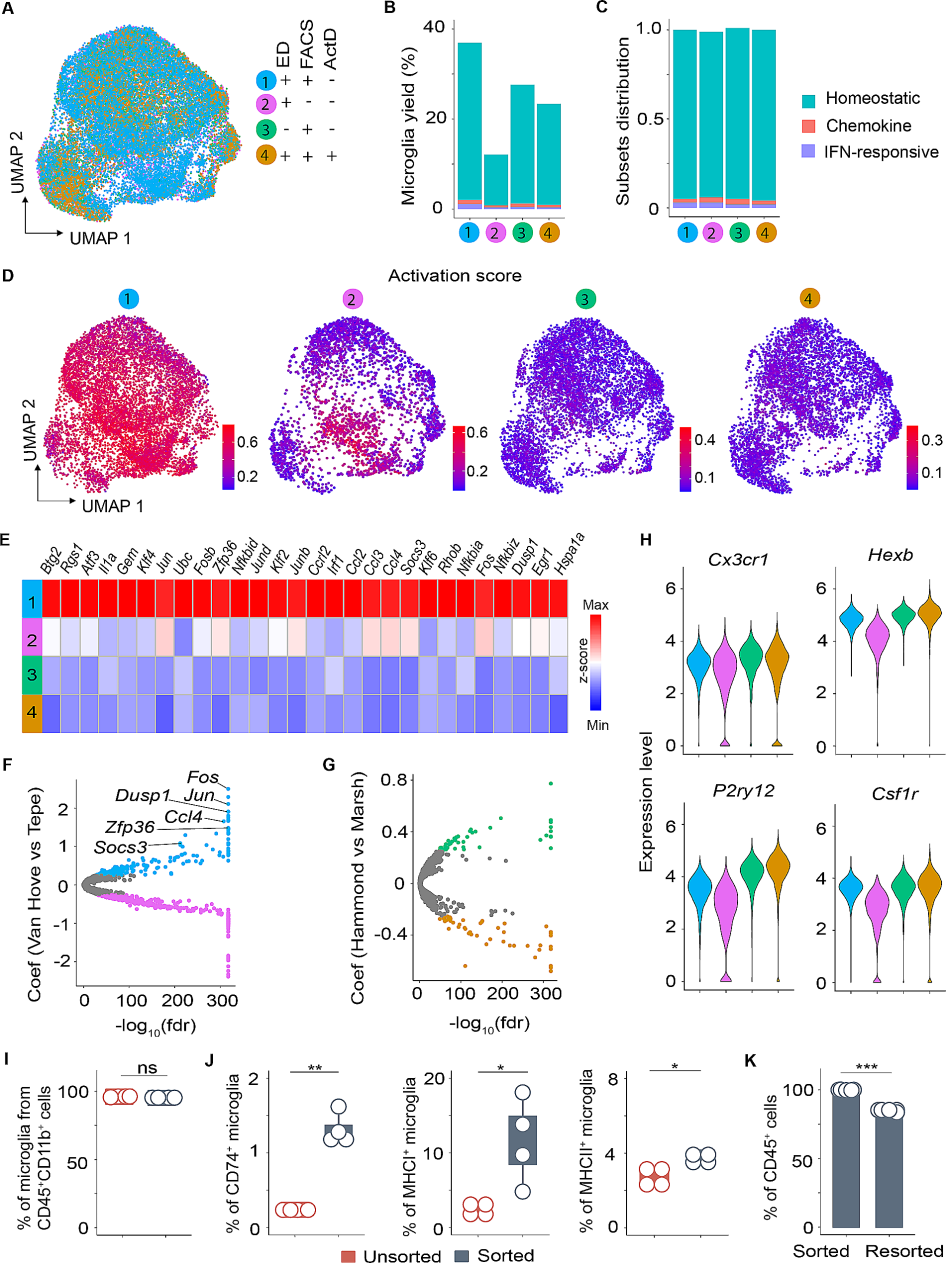
Meta-analysis of microglia identifies profound effects of cell sorting on microglial transcriptional profiles. **A**, UMAP plot showing clustering of microglia from Von Hove et al. [23], Tepe et al. [26], Hammond et al. [31], and Marsh et al. [27] after QC and data filtering using Harmony integration. The cells are colour-coded according to the legend shown on the right. **B**, Bar plot depicting microglia yield in percentages across the integrated dataset. **C**, Distribution of microglia subsets per dataset displayed on bar plot. **D**, UMAP plot displaying UCell score distribution for the cell activation signatures within the microglia-integrated dataset stratified by experimental setup. **E**, Heatmap showing differential expression of cell activation genes across the four datasets. **F**, Volcano plot displaying differentially regulated genes in microglia isolated by enzymatic dissociation and cell sorting (blue) versus cell sorting without enzymatic dissociation (magenta). Genes with coefficient of <|0.25| and FDR > 0.05 are colour coded in grey. **G**, Volcano plot highlighting differentially regulated genes in microglia isolated by cell sorting without enzymatic dissociation (green) relative to cell sorting with enzymatic dissociation (umber). **H**, Violin plot generated from the integrated dataset displaying characteristic microglial marker genes across the experimental setup. **I**, Plot of percentages of microglia from CD45^+^CD11b^+^ cells in unsorted and sorted fractions. **J**, Boxplot with dotplot overlays displaying percentages of microglia expressing CD74, MHC I and MHC II in unsorted and sorted cells. **K**, Barplot depicting percentages of CD45^+^ cells after sorting and resorting (*n* = 4 mice per group, two-tailed paired t test; * *p* < 0.05, ** *p* < 0.01, *** *p* < 0.001)

After quality control, data integration, and regressing out the technical noise, single-cell transcriptomic profiles for 25,406 putative microglia cells were retained for analysis. Visual representation of integrated data projected onto UMAP revealed that microglia consolidated into one single cluster across all the analyzed experimental setups (Fig. 3A). As expected, using unsupervised clustering, the integrated data revealed three microglial clusters (Fig. S3A), verifying the earlier observation that homeostatic microglia are transcriptionally heterogeneous [27, 32, 33]. Depending on the experimental protocols applied by the authors, we observed qualitative differences in the percentages of microglia identified amongst the analyzed cells (Fig. 3B). Of note, we found that microglia isolated by enzymatic digestion without enrichment by flow cytometric sorting yielded lower abundance of microglia amongst the analyzed cells than other experimental approaches that involved cell enrichment steps. Nevertheless, the composition of the microglia subsets remained relatively similar in all the analyzed datasets (Fig. 3C). These observations indicated that the distribution of microglia subsets is not significantly affected by the different experimental approaches.

To examine the presence of activation signatures in the integrated datasets, we performed gene module scoring using activation-induced signatures. By projecting this score onto UMAP, we found that microglia isolated by standard enzymatic tissue dissociation coupled with flow cytometric sorting were enriched for cells that were positive for activation signatures (Fig. 3D and Fig. S3B). Although to a lesser extent, microglia isolated by sorting without enzymatic dissociation also displayed cell activation signatures. In contrast and consistent with previous data [27, 31], we found that microglia isolated under ice-cold conditions or in the presence of transcriptional inhibitors exhibited de-enriched cell activation scores (Fig. 3D and Fig. S3B).

We further examined expression levels of these activation-induced signatures at the gene level. In concordance with gene module scoring, microglia isolated by enzymatic digestion and sorting showed significant upregulation of immediate early genes (*Jun*,* Egr1*), stress-induced genes (*Hspa1a*,* Dusp1*) as well as immune-related genes (*Ccl4*,* Nfkbiz*) (Fig. 3E). Of note, these genes were abrogated in microglia isolated under ice-cold conditions or treated with transcriptional inhibitors during the tissue dissociation step (Fig. 3E). Furthermore, we observed negligible differences in the expression levels of these activation signatures between the different microglia subsets (Fig. S3C).

A key aspect of nearly all scRNA-seq experiments involves dissociation of solid tissues, enzymatically or mechanically, to release individual cells and the use of sorting to enrich microglia in a final step. We wondered whether these two entwined cell isolation steps could contribute to cell activation. To address this, we performed differential state analysis on the integrated microglial datasets using the MAST package [34]. To determine whether the sorting procedures induced aberrant microglial signatures, we performed differential analysis on microglia isolated by enzymatic dissociation with or without cell sorting (Van Hove et al. versus Tepe et al.). Interestingly, we observed that cell activation genes (*Fos*,* Jun*,* Dusp1*,* Ccl4*,* Zfp36*,* Socs3*) were significantly higher expressed (Coeff > 0.25 and FDR < 0.05) in microglia isolated by enzymatic tissue dissociation and cell sorting than in microglia isolated by enzymatic dissociation without cell sorting (Fig. 3F and Fig. S3D).

To further assess the impact of enzymatic digestion, we carried out differential analysis of microglia isolated by cell sorting without enzymatic dissociation versus cell sorting with enzymatic dissociation (Hammond et al. versus Marsh et al.). Surprisingly, we found complete absence of activation-induced gene signatures in microglia that were isolated by either cell sorting without enzymatic digestion or cell sorting with enzymatic digestion (Fig. 3G and Fig. S3D). Arguably, this implies that the use of cell sorting rather than enzymatic dissociation is the key factor inducing aberrant cell activation signatures in microglia. Furthermore, post-hoc analysis revealed that irrespective of the experimental differences that were adopted by the authors, expression levels of the canonical microglia markers *Cx3cr1*, *Hexb*, *P2ry12*,* C1qa*,* Siglech*,* Csf1r*, and *Trem2* were rather similar, highlighting that these markers are overall stably expressed in microglia (Fig. 3H and Fig. S3E).

To corroborate the impact of cell sorting on microglia expression profiles on the proteomic level, we isolated brain-resident immune cells by standard enzymatic tissue dissociation and analyzed the expression of cell surface markers by flow cytometry on unsorted and CD45^+^ sorted cells. Quantitative analysis revealed that the percentage of microglia from the CD45^+^CD11b^+^ population was similar in unsorted and sorted cells (Fig. 3I; gating strategy in S3G). Nevertheless, we observed a significant increase in the percentage of microglia expressing CD74, MHC I and MHC II in the sorted fraction relative to the unsorted cells (Fig. 3J and Fig. S3H). Interestingly, after a second sorting of CD45^+^ cells, we detected a significant decrease in the absolute number of CD45^+^ cells (Fig. 3K), which can be explained by cell loss due to cell death that was induced by the cell sorting procedure. This control experiment highlighted that shear stress or traumatic injury is induced during the cell sorting procedure, which might be a potential inducer of transcriptional and proteomal alterations of microglia.

### Differential state analysis reveals that *bona fide* microglial signatures are confounded by ex vivo induced artefacts

**Fig. 4:**
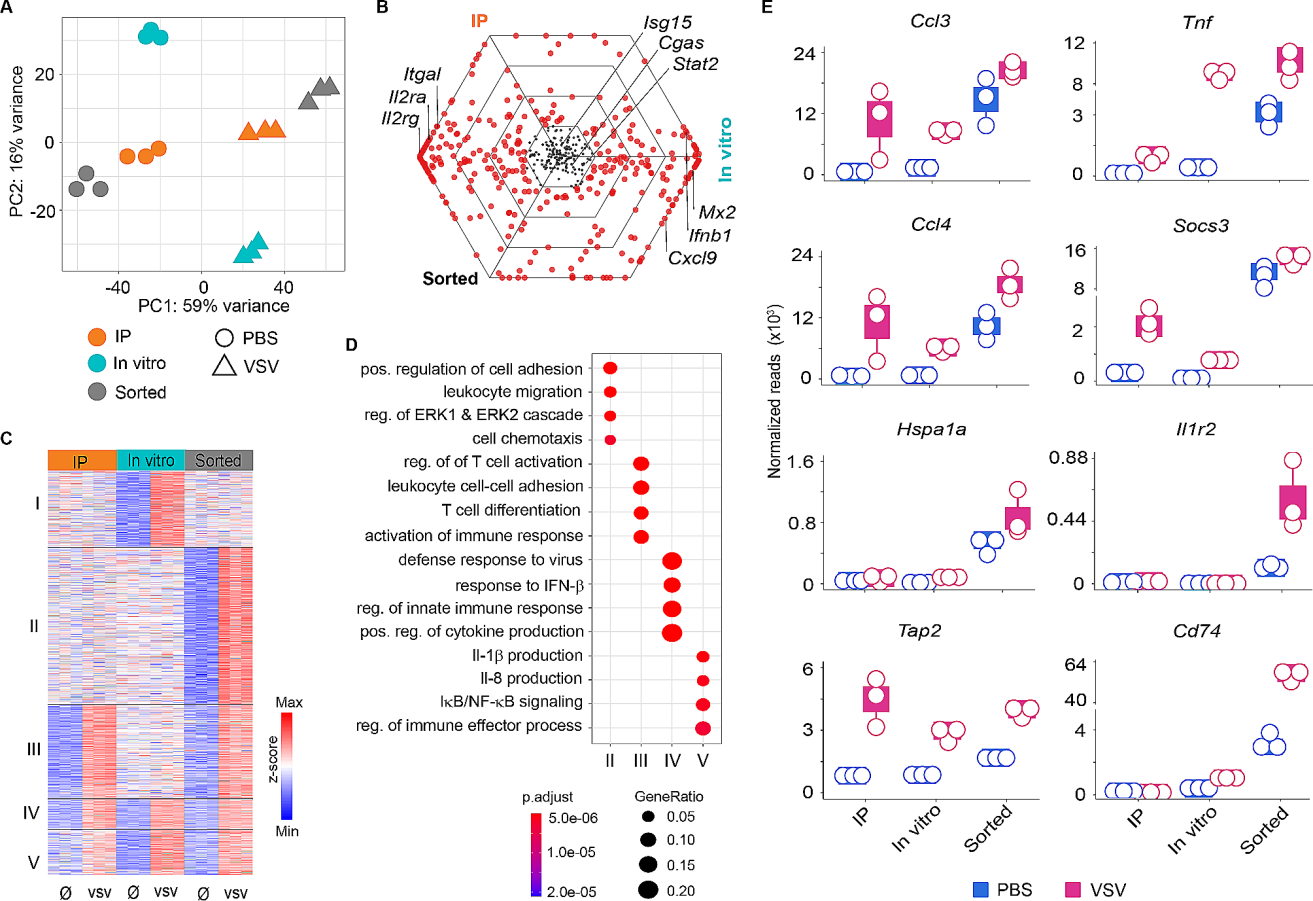
Microglia *bona fide* signatures are masked by microglial activation-induced signatures. **A**, PCA displaying clustering of samples derived from IP, in vitro, and sorted microglia after VSV treatment. Each dot represents a single mouse. **B**, Triwise plot displaying expression strength (log-fold change) of overlapping genes in IP, in vitro, and sorted microglia. The internal hexagon corresponds to genes with the same expression in all three samples. Genes lying on hexagonal gridline have the same maximal fold change between any two pairwise comparisons. **C**, Heatmap clustered by k-means clustering, comparing VSV-treated with mock controls from IP, in vitro, and sorted microglia samples, revealing five clusters. **D**, Top five gene ontology terms associated with differentially regulated genes from heatmap clusters in Fig. 4C. **E**, Boxplots with dotplot overlays showing normalized gene counts of selected genes related to microglial activation regulated upon VSV challenge in IP, in vitro, and sorted samples (for additional details see caption of Fig. 1)

Beyond post-hoc analysis of large-scale datasets, we next assessed whether differential analysis would reveal aberrant activation signatures on VSV infected samples that were processed concurrently by same methods. To address this, we performed transcriptomic and translatomic analyses of microglia derived from IP, in vitro, and sorting after either PBS treatment or VSV infection. By controlling several batches as covariates, principal component analysis (PCA) showed a clear separation of IP, in vitro and sorted microglia (Fig. 4A). Notably, VSV-infected samples clearly segregated from uninfected controls as shown by PC1 accounting for 59% variance, suggesting the induction of substantial transcriptional changes of microglia upon virus infection. Among the VSV-infected groups, in vitro samples largely segregated from IP and sorted samples, whereas this effect was much less pronounced when IP and sorted samples were compared. This highlighted that microglia from different experimental setups display distinct expression profiles upon virus infection. On the other hand, uninfected samples from IP, in vitro and sorted microglia clustered separately (Fig. 4A). It could seem counterintuitive that IP, in vitro or sorted microglia from uninfected samples were so dissimilar, considering that all microglia were analyzed at homeostatic state. However, this observation indicated that adoption of different experimental setups for transcriptional profiling of microglia have different confounding effects.

We performed differential analysis on VSV-infected samples and their respective controls. By applying selection criterion of log_2_-fold change >|1|, padj < 0.05, we identified 2332, 3063 and 3110 genes that were differentially expressed upon VSV challenge in IP, in vitro, and sorted microglia, respectively, and 492 differentially expressed genes being shared by all three setups (Fig. S4A). Of the 492 consensus genes, 20% were induced at similar levels as indicated by the log-fold changes of the expression in IP and sorted samples (Fig. S4B). These genes included interleukins such as *Il2rg*, *Il2ra* (Fig. 4B). Correspondingly, we identified 15% of the consensus genes that had a maximal fold change in in vitro microglia (Fig. S4B). These genes comprised *Mx2*, *Ifnb1*, and *Cxcl9* amongst others (Fig. 4B). Interestingly, interferon-stimulated genes (ISGs) such as *Isg15*, *Cgas*, and *Stat2* were expressed at similar levels in all three microglia samples (Fig. 4B). Unbiased k-means clustering of differentially regulated genes revealed the presence of five different clusters (Fig. 4C). Cluster I and II comprised genes that were upregulated upon VSV infection in in vitro and sorted microglia. Cluster III was represented by genes that were upregulated in IP and sorted microglia (Fig. 4C). Notably, cluster IV consisted largely of genes that were upregulated under all three experimental conditions, while cluster V comprised genes that were abundantly expressed in in vitro and sorted samples, and that were expressed to a lesser extent in IP samples (Fig. 4C). To corroborate the functional phenotype associated with the identified cluster-specific genes, we performed pathway analysis (P value cutoff < 0.05) using GO gene sets. Genes in cluster I did not attain the cutoff criteria to infer associated pathways. Nevertheless, cluster II exclusively expressed genes associated with positive regulation of cell adhesion, leukocyte migration, and cell chemotaxis (Fig. 4D). Regulated genes in cluster III are involved in T-cell differentiation, regulation of T-cell activation, and activation of immune responses (Fig. 4D). Notably, cluster IV and V contained genes that are implicated in responses to virus, positive regulation of cytokine production, and pro-inflammatory responses such as interleukin-1 (IL) beta, IL-8 production, and NF-kB signaling (Fig. 4D). Importantly, the homogenous expression of genes associated with these pathways reinforces the idea that microglia are key meditators of inflammatory responses during VSV infection. Intriguingly, we observed that the microglial responsiveness to VSV infection was not affected by the experimental approaches applied (Fig. 4D).

The identification of GO terms corresponding to (i) leukocyte migration, (ii) the ERK1 and ERK2 cascade, and (iii) cell chemotaxis in cluster II (Fig. 4D) prompted us to ask whether the identified aberrant signatures in sorted microglia could be misinterpreted as being differentially expressed upon exposure to VSV. To address this, we sorted and filtered genes based on the aforementioned GO terms. Remarkably, genes associated with pro-inflammatory signaling (*Ccl3*,* Tnf*,* Ccl4*,* Il1r2*), antigen processing and presentation (*Tap2*,* Cd74*), oxidative stress (*Hspa1a*), and IFN-induced response (*Socs3*) were highly elevated in sorted microglia relative to in vitro or IP microglia under basal conditions, and were further upregulated in sorted microglia after VSV infection, albeit to different extents in in vitro and IP microglia (Fig. 4E). Collectively, these data highlighted that activation-induced signatures are masked as being “differentially expressed” in samples with disease status, thus jeopardizing attempts to delineate *bona fide* microglia disease signatures.

### Microglia develop transcriptional heterogeneity in response to VSV-induced encephalitis

**Fig. 5:**
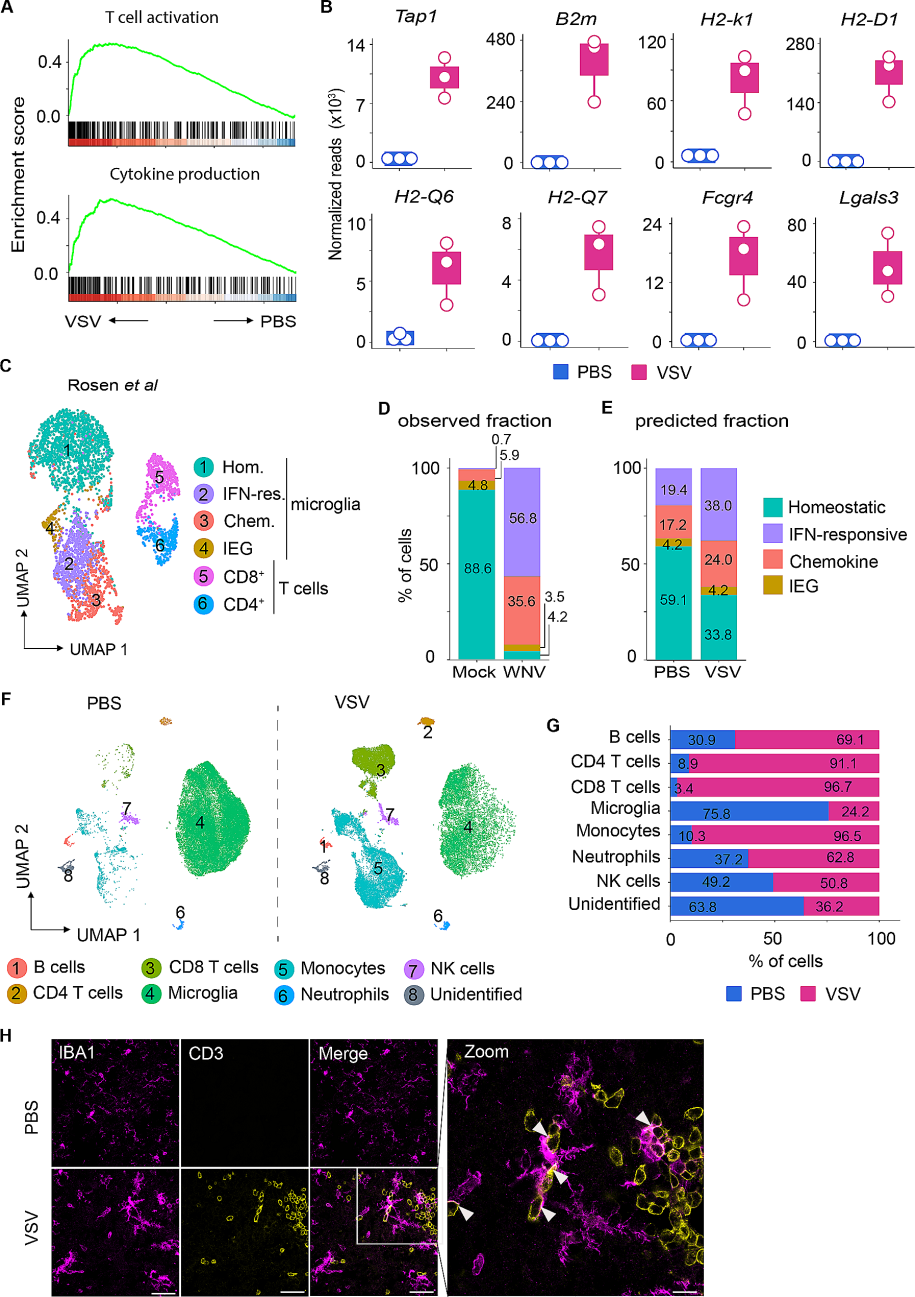
VSV-induced inflammation causes significant changes in the microglial transcriptome. **A**, GSEA displaying top two enriched GO terms associated with differentially expressed genes in the microglia translatome. **B**, Boxplots with dotplot overlays representing normalized gene counts of selected genes associated with above enriched pathways in microglia translatome (additional details in caption of Fig. 1). **C**, UMAP representation of scRNA-seq analysis of 4,010 cells from brain highlighting major myeloid and T-cell subsets. **D**, Barplot representing the observed proportion of microglia clusters in mock and WNV-infected groups. **E**, Barplot highlighting the in-silico predicted proportion of microglia subtypes in PBS and VSV-infected samples. **F**, UMAP plot showing segregation of immune cell subtypes within the brains of uninfected and VSV-infected mice at 6 dpi (*n* = 3 mice per group). **G**, Barplot representing percentages of immune cell composition within the brains of PBS and VSV-infected mice. **H**, Representative images of OB immunolabeled for IBA1 and CD3. VSV-infected OB show influx of CD3^+^ cells on day 6 after infection. The white arrows denote IBA1^+^ cells engaged with CD3^+^ cells (*n* = 3 mice per group, *N* = 2; combined data; Scale bar 200 μm)

Thus far, our data demonstrated that the RiboTag approach not only allows for the analysis of microglia-associated mRNA expression profiles, but also precludes non-specific microglia activation and concurrent upregulation of early response genes [35]. We therefore focused on IP samples for in-depth profiling of microglia to assess the nature of innate response to viral infection. We identified 2037 genes (1322 up- and 715 downregulated) that were differentially expressed in response to VSV infection (Fig. S4C). Correlation of functional relevance of the identified genes using gene set enrichment analysis (GSEA) demonstrated significant enrichment of genes that are mostly associated with T-cell activation and cytokine production (Fig. 5A). Among these, genes encoding for the MHC complex (*Tap1*,* B2m*,* H2-Q6*,* H2-K1*,* H2-D1*), co-stimulation (*Cd86*), Fc receptor (*Fcgr4*), and phagocytosis (*Lgals3*) showed enhanced expression in microglia derived from VSV-infected animals (Fig. 5B). Given that MHC I expression usually is very low or even undetectable on cells in the CNS under homeostatic conditions, the pronounced expression of MHC I on microglia from VSV-infected mice indicated an inflammatory status of the cells that is presumably associated with enhanced antigen cross-presentation. To explore this observation beyond the translatomic level, we performed re-analysis of scRNA-seq data of CD45^+^ cells isolated from the brain of mice that recovered from West Nile virus (WNV) infection and the respective mock controls [33]. Notably, infection with an attenuated WNV strain induces self-limiting brain inflammation accompanied by microglia activation that is similarly observed during VSV-induced encephalitis. In accordance with Rosen et al. [33], we identified six distinct clusters comprising four microglia subsets (homeostatic, immediate early gene, chemokine, and IFN responsive clusters) and two T cell subsets (CD4^+^ and CD8^+^ T cells) (Fig. 5C and Fig. S4D). Closer examination of the microglia clusters for the expression of genes that are associated with MHC complexes and phagocytosis revealed pronounced expression of the genes *Tap1*,* B2m*,* H2-Q6*,* H2-Q7*,* H2-K1*,* H2-D1*,* Fcgr4*, and *Lgals3* in IFN-responsive microglia, whereas these genes were induced to a lesser extent in chemokine microglia, emphasizing that microglia exhibit transcriptional heterogeneity in response to viral infection (Fig. S4E). In transcriptomic analyses of whole tissues, RNAs from all cells are analyzed *en masse* and therefore cellular heterogeneity can be missed. To circumvent this and to analyze distinct microglial subtypes, we implemented an in-silico deconvolution method that leverages scRNA-seq data to infer cell subtype proportions in bulk samples [36]. Since deconvolution methods often underperform when used to compare the proportions between different cell types [37, 38], we selected microglia clusters from the above scRNA-seq data [33] as a reference. Within the computed proportions of scRNA-seq microglial sub-clusters in the mock control or in WNV-infected samples (Fig. 5D and Fig. S4D), we predicted significant abundance of homeostatic, immediate early gene (IEG), chemokine and IFN responsive microglia subtypes in our bulk microglial translatome (Fig. 5E). Intriguingly, 19.4% and 17.2% of the microglia translatome from the PBS control were enriched for IFN-responsive and chemokine microglia subtypes, respectively, which under conditions of VSV infection increased to 38.0% and 24.0%, respectively (Fig. 5E). Unexpectedly, the translatome of PBS microglia was highly enriched for homeostatic microglia (59.1%), whereas this subset was decreased to 33.8% in VSV infected microglia, suggesting elimination or phenotypic transformation of activated microglia during viral encephalitis. As expected, the microglia subtype expressing immediate early genes was present at low abundance in PBS and VSV-infected microglia translatome (4.2%) (Fig. 5E). Taken together, the expression deconvolution analysis revealed the presence of microglia subtypes at different abundancies in mock vs. VSV infection at the bulk RNA level.

### VSV-induced encephalitis is associated with massive T-cell infiltration and close interaction of T cells with microglia

Recent studies demonstrated that VSV titres in the OB are controlled by day 6 post-infection, which coincides with activation of microglia and influx of monocytes [4]. Although the immune cell dynamics are very complex in the infected brain and can vary significantly during the course of infection, we previously reported robust recruitment of CD45^+^ cells as early as day 4 after infection, and that infiltration further exacerbated by day 6 after infection [39]. To provide a snapshot of the diversity of infiltrating immune cells in the brain, we performed flow cytometric analysis of immune cell populations in the brain of C57BL/6 mice 6 days after VSV infection. For unbiased analysis of the samples, we gated on CD45^+^ cells and performed clustering and dimensionality reduction by using UMAP (Fig. S3G). This approach revealed the presence of 7 distinct clusters including lymphocyte populations comprising CD4^+^ and CD8^+^ T cells, B cells, NK cells, granulocytes (neutrophils), and myeloid subsets such as monocytes and microglia (Fig. S4F). However, a minor population of an undefined cluster was detected that corresponded with cells for which the analyzed marker combination did not allow further specification (Fig. S4F). Nevertheless, segregation of samples based on the treatment revealed clear stratification of immune cell populations (Fig. 5F). The proportions of cell types varied considerably across the treatment groups with high percentages of microglia detected in the control group (75.8%) that declined to 24.2% after VSV infection (Fig. 5G). In VSV-infected samples we detected dramatic influx of monocytes, neutrophils, B cells, NK cells, and T cells when compared with PBS treated controls. Specifically, in the mock-treated group, the percentage of CD8^+^ and CD4^+^ T cells amongst CD45 + cells was low with 3.4% and 8.9%, respectively, whereas under conditions of VSV-infection a profound increase of both CD8^+^ (96.7%) and CD4^+^ (91.1%) T cells was detected (Fig. 5G). These data demonstrated that the uninflamed CNS contained very few adaptive immune cells, whereas following VSV infection, T cells were massively recruited into the brain.

Our translatomic analysis revealed that, unlike homeostatic microglia, VSV-experienced microglia express MHC and co-stimulatory molecules, which would allow the microglia to closely interact with infiltrating T cells. To further analyze the microglia-T cell interaction on the protein level, we inoculated C57BL/6 mice intranasally with either PBS or VSV and performed histological analysis of the OB at 6 dpi. As similarly detected by flow cytometry, histological analysis of OB samples labeled for IBA1 and CD3 revealed a dramatic influx of CD3^+^ cells into the OB of infected mice when compared with PBS treated controls (Fig. 5H). Interestingly, CD3^+^ and IBA1 + cells were located in close proximity, suggesting that T cells interacted with the myeloid cells. Dense IBA1^+^ conglomerates being surrounded by CD3^+^ cells further supported the hypothesis that microglia present antigen to the T cells. In summary, our histological data showed that CNS infiltrating T cells enter into close interaction with microglia in the virus infected CNS.

## Discussion

Microglia are resident myeloid cells of the CNS that are implicated in numerous physiological and pathological processes [9, 40, 41]. Here, we exploited several approaches, including in vitro generated microglia, the myeloid cell-selective RiboTag approach, and cell sorting of brain resident myeloid cells, to decipher *bona fide* signatures of microglia under conditions of health and viral encephalitis. All three models revealed a common set of core microglial transcripts, highlighting the suitability for the applied approaches to investigate microglia biology. However, we discovered that sorted microglia showed aberrant transcriptional alterations that were associated with the cell sorting procedure. Interestingly, such signatures we detected also in large-scale transcriptomic atlases that are commonly used for reference-based cell type annotations. Analysis of the translatome of virus-experienced microglia revealed pronounced expression of genes encoding for components of the MHC complex and co-stimulatory receptors, implying that microglia exhibit an enhanced potency to present antigen. Cytometric analysis revealed the massive influx of peripheral immune cells into the infected brain, including lymphocytes and myeloid cells, by day 6 after infection. At the protein level, we uncovered that infiltrating T cells surrounded the dense conglomerates of microglia, suggesting that T cells intimately interact with activated microglia.

A major challenge in transcriptomic analysis of microglia is contamination of sorted cell preparations with other CNS-resident cells that might occur during conventional whole-cell preparation procedures [42, 43]. Our results demonstrated efficient enrichment of myeloid cells in IP and sorted microglia when compared with the input cells (Fig. 1C-E), as indicated by the absence of astrocytic, neuronal, and oligodendrocytic markers in the RNA-seq data. Although these data emphasize the specificity of the CX3CR1-Cre^ERT2+/−^ model in inducing efficient recombination in myeloid cells, we cannot rule out targeting of other long-lived non-parenchymal brain macrophages. Although there are other mouse lines showing inducible Cre expression in microglia such as HEXB-Cre^ERT2^ [14], TMEM119-Cre^ERT2^ [15] and P2RY12-Cre^ERT^ [2,13] as well as binary Cre models [44], they are less efficient in recombining all microglia [45] and their utility in the context of infectious and non-infectious encephalitis models remains to be proven. The absence of core gene signatures of other brain-resident cell types in in vitro cultivated microglia indicated that also this approach was appropriate and effective in generating highly pure preparations of microglia that are suitable for transcriptomic studies.

Comparison of the gene expression profiles derived from IP, in vitro, and sorted microglia revealed commonalities and differences in gene expression. Consistent with prior reports [17], we detected exclusive expression of several *bona fide* stress-regulators (*Zfp36*,* Dusp1*,* Jun*,* Ccl4*) in sorted microglia (Fig. 2C and S1A). Notably, Zinc finger protein 36 (*Zfp36*) is involved in regulating immune responses through mRNA destabilization and alternative splicing [46], whereas dual-specificity phosphatase-1 (*Dusp1*) is a key component in regulating anti-inflammatory responses that is expressed in response to stressors, such as heat shock or oxidative damage [47]. Kinetics evaluation of chemokine ligand-4 (*Ccl4*) previously revealed this gene to be expeditiously expressed following stimulation [48], and as such, it is likely that detection of this gene in sorted microglia is an ex vivo activation artifact. Noteworthy, given the prevalent nature of *Dusp1*, *Zfp36*, and other genes identified here (Supplemental Table 1), these are useful proxy markers for evaluating aberrantly activated microglia. A long-standing concern is whether such ‘spurious’ signatures are unique to microglia, or whether they are also prevalent in peripheral immune cells. Our post-hoc analysis on published scRNA-seq datasets revealed a widespread existence of aberrant transcriptional signatures in CNS-resident myeloid cells and non-CNS cell types, calling into question whether findings reported in those studies fully reflect the in vivo status of the analyzed cells [23, 29, 30]. In an era when biologists increasingly rely on scRNA-seq to discern cellular functions, ex vivo transcriptional alterations constitute a major technical challenge for many studies. Moreover, these spurious signals appeared to be present not only in study-specific datasets but also in reference atlases, underscoring the need for benchmarking to assess the quality and accuracy of single-cell reference atlases. Furthermore, with the emergence of large-scale consortia such as Human Cell Atlas project [49] and murine cellular atlases [50] that are aiming at establishing comprehensive transcriptional maps for multicellular tissues, the development of approaches that compensate or avoid such transcriptional biases is crucial.

A critical step in transcriptomic studies is the isolation of intact single cells from tissue with the highest possible purity. The isolation of microglia represents a major challenge. Due to their elaborate and long processes entwined with other cell types within the CNS, separation of microglia from neighboring cells requires extensive tissue degradation, which increases the likelihood of rupturing the microglial cell membrane. Notwithstanding, enzymatic digestion or mechanical dissociation of the brain tissue combined with cell sorting is a standard method for obtaining sufficiently high cell numbers of microglia that are required for omics applications [23, 51]. We found that microglia yields were lower using enzymatic digestion protocols without cell sorting (Fig. 3B), re-emphasizing the importance of cell enrichment in transcriptomic analyses of low abundant cell types of the CNS such as microglia. However, we uncovered that cell sorting is a harsh procedure that elicits fundamental alterations of the microglial transcriptome or proteome. Specifically, we found that hydrodynamic stress and/or traumatic injury encountered during cell sorting are key inducers of ex vivo microglial activation. Such aberrant signatures not only distort baseline transcriptional profiles of microglia, but also confound expression patterns that are truly induced by pathological insults. Indeed, we detected elevated expression of genes associated with pro-inflammatory signaling (*Ccl3*,* Tnf*,* Ccl4*,* Il1r2*), antigen presentation (*Tap2*,* Cd74*), oxidative stress (*Hspa1a*) and IFN-induced response (*Socs3*) which are concealed as being ‘differential’ in sorted microglia upon VSV infection (Fig. 4E). Induction of *Cd74*,* Ccl3*,* Tnf,* and *Ccl4* genes that are well documented in neuroinflammation and disease states [52–54], indicates microglia switching from a homeostatic to a more inflammatory state. This is in contrast to the view that microglia which are polarized towards an activation state (i.e., response to disease) are less responsive to new stimuli (i.e., cell isolation) [27]. Therefore, it appears that ex vivo activation signatures associated with cell sorting are confounded by biological treatment. In conclusion, our results underscore the need for recognizing cell sorting-related signatures, which have substantial implications in obscuring the identification of true biological states of cells in health and disease. Moreover, we re-emphasize the importance of validating RNA-seq results from cell sorted tissues to disentangle these aberrant signatures from *bona fide *in vivo derived signatures of tissue resident cells such as microglia that are particularly sensitive to removal from their in situ microenvironment.

Efforts to circumvent cell-specific transcriptional biases are indispensable not only for enhancing the comparability of the findings, but also to avoid data misinterpretation and drawing false conclusions. Cold isolation protocols and transcriptional inhibitors have both emerged as potential strategies for preserving in vivo cellular signatures [27, 31]. In line with previous studies [27, 31], we found that scRNA-seq of microglia generated with inclusion of transcriptional inhibitors, or processing tissue under ice-cold conditions, invariably diminishes aberrant microglial activation signatures. Although transcriptional blockers are effective in preventing *de novo* gene expression, it must be highlighted that they do not prevent degradation of pre-existing RNAs [55]. Thus, the expression of genes such as *Ubc*,* Rac1*, and *Tgfb1* under conditions of Actinomycin D treatment most probably reflects the activation status of microglia (Fig. S3F). Unfortunately, the above-mentioned strategies can have unintended consequences on cellular functions beyond gene expression. By blocking protein synthesis, transcriptional inhibitors can disturb normal cellular processes that potentially impact cell viability, proliferation, and metabolism. Moreover, transcriptional inhibitors were reported to interfere with the ability of cells to respond to stimuli, impairing their capacity to mount appropriate immune or stress responses [56]. Several studies have demonstrated that exposing mammalian cells to sub-physiological temperatures invokes a coordinated cellular response involving the induction of cold-shock proteins and the modulation of translation, metabolism, cell cycle, and the cytoskeleton [57]. Therefore, we strongly urge the selection of appropriate cell isolation methods to favor accurate biological profiling in studies involving stress-responsive immune cells.

Considering the immense effort put into the generation of transcriptomic atlases, it is essential to retrospectively attenuate or completely remove ex vivo activation signatures from existing datasets. Indeed, unprecedented numbers of computational strategies that entail complex machine-learning algorithms to detect and remove ex vivo activation genes have been developed [58]. However, this comes with major caveats: Firstly, the expression levels and dynamics of different activation genes vary substantially between cell types and brain regions [59]. Thus, pre-selecting one or few genes without a systematic survey may limit the detection of activated cell populations. Secondly, computational removal of ex vivo activation genes can eliminate ‘real’ signals thus jeopardizing studies that intend to examine biologically relevant acute gene expression changes. Lastly, ex vivo transcriptional changes may not always be restricted to early response genes and determining which genes to exclude from the analysis could be difficult or impractical.

Using the RiboTag approach, we discerned cell-type specific transcripts that were actively translated by microglia, herein referred to as ‘translatome’. Since the RiboTag approach inherently relies on the enrichment of ribosomes from selected cell types rather than the purification of cell types from whole tissues [16, 60], we incorporated the concept of a log2-fold change as enrichment factor between the IP and input to assess the cell-type specificity of a given transcript. As previously described by Sanz et al. [16], we adopted the stringent cut-off of 1-fold higher or lower for transcript enrichment or depletion, respectively. With this approach, transcripts that are enriched are more likely specifically expressed within the cell type of interest, whereas transcripts that are depleted are more likely to be expressed in other cell types contained within the tissue. Of note, by direct comparison of input, IP, in vitro, and sorted microglia, our study highlighted unique signatures of homeostatic microglia as being represented in cluster II and III (Fig. 1B). Specifically, unlike scRNA-seq or conventional bulk RNA-seq, RiboTag does not require cell dissociation and/or cell sorting [16]. Therefore, this unified set of genes accurately reflects in situ microglia profiles. Indeed, determining the degree of ex vivo activation artefacts has been challenging because the field lacked a resting cell type-specific reference, i.e., absence of any ex vivo activation as a comparator in building an accurate and complete CNS atlas. It is conceivable that the microglial translatome identified here provides a comparator to deconvolute ex vivo artefacts in publicly available scRNA-seq datasets. More broadly, the RiboTag approach represents a unique methodology to discover transcripts expressed by rare and difficult-to-isolate cell populations such as tissue macrophages and other resident leukocyte-derived cells.

In the context of viral infections, microglia are often the first responders that are critically needed to protect the host against lethal encephalitis [61, 62]. We exploited the RiboTag approach to illuminate microglia expression profiles in a more complex inflammatory scenario than neuroinflammation induced by intraperitoneal LPS administration [17]. It is worth pointing out that the choice of an infection model such as intranasal VSV infection relies mainly on the fact that VSV infection causes a high infiltration of lymphoid and myeloid cells into the brain. Furthermore, by fate mapping experiments we have previously observed that several microglia can shift into the CD45^high^ gate of monocytes [4]. By analysis of the translatome of microglia, we uncovered the induction of gene signatures implicated in T-cell activation and cytokine responses upon VSV infection (Fig. 5A). The very abundant expression of genes encoding elements from MHC I and II complexes, cell activation, and Fc receptors (Fig. 5B) further supported the concept that microglia are involved in the regulation of T-cell mediated antiviral responses within the CNS. Previous studies reported synergistic roles of T cells and microglia in CNS viral infection [63, 64]. Depletion of CD4^+^ and/or CD8^+^ T cells was shown to impede VSV containment within the OB and to promote development of lethal encephalitis in infected mice [39, 63], suggested that T cells exert antiviral functions during CNS infection. Furthermore, microglia depletion was reported to accelerate encephalitis, and alter T cells dynamics as well as cytokine responses during viral CNS infection [64, 65]. Cytokine signaling has different effects depending on the cell type that receives the signal. The IFN-γ released by infiltrating T cells was shown to induce microglia activation, as evidenced by upregulation of MHC II on these cells, which is normally expressed at lower levels [66]. However, Klein et al. [67] and Garber et al. [66] demonstrated that in the context of WNV and Zika virus encephalitis prolonged IFN-γ secretion that persists beyond viral clearance leads to microglia-mediated synaptic stripping and memory impairment. Since CNS entry of immune cells is tightly regulated [68], the influx of monocytes, neutrophils, B cells, and NK cells affirms their importance in controlling viral infection. However, the interplay between these cells and microglia remains to be in depth investigated.

Following VSV infection, IBA1^+^ cells were surrounded by, and some were in direct contact with, CD3^+^ cells (Fig. 5H) indicating close T cell-microglia interaction. Given that microglia are antigen presenting cells, it is conceivable that infiltrating T cells recognize cognate viral peptides displayed on the surface of microglia and that this way T cell-mediated immunity is orchestrated in the brain. Indeed, Moseman and colleagues [63] observed an overall decline in the calcium flux of cytotoxic T lymphocytes in mice deficient of MHC I in the CNS resident compartment, supporting the notion that antigen presentation to VSV-specific CD8^+^ T cells primarily relies on radiation resistant brain cell populations such as microglia. Considering the dramatic influx of CD4^+^ T cells in the inflamed brain (Fig. 5G) it is likely that MHC II-positive microglia take up viral antigens and present antigen fragments to infiltrating CD4^+^ T cells. Recent studies reported a critical role of CD4^+^ T cells in viral infection of the CNS. For instance, Wheeler et al. [64] revealed diminished virus-specific CD4^+^ T cell responses in the absence of microglia and decreased survival upon mouse hepatitis virus (MHV) infection of mice. Nevertheless, in CNS auto-immune disorders, microglial MHC II antigen presentation appears obsolete for the establishment of EAE [69, 70].

In conclusion, the RiboTag approach reveals accurate *bona fide* signatures of homeostatic as well as viral encephalitis microglia. Such signatures serve as a comparator that can be used to re-evaluate the presence of aberrant microglial signatures that are associated with cell isolation procedures in other bulk RNA-seq or scRNA-seq datasets. We further illuminated transcriptional changes associated with virus-induced inflammation in microglia and deciphered microglia-T cell interaction in shaping distinct antiviral response of the brain. Taken together, this study provides comprehensive molecular insights in the biology of microglia and their interplay with peripheral innate and adaptive immune cells, which makes it possible to identify novel genes, pathways, and regulatory factors that are critical for microglia functions in health and disease.

## Methods

### Mouse lines

Experiments to investigate functional traits of microglia were conducted with C57BL/6J that were referred to as wild type (WT), CX3CR1-Cre^ERT2+/−^ [71] and Rpl22^HA/HA^ [16] mice on the C57BL/6 background. To selectively label ribosomes of microglia in vivo, CX3CR1-Cre^ERT2+/−^, which express a Cre-ERT2 fusion protein under the CX3CR1 promoter, were crossed with Rpl22^HA/HA^, in which exon 4 of the Rpl22 gene is floxed and followed by a modified exon 4 to which a HA tag is fused. Upon tamoxifen injection of CX3CR1-Cre^ERT2+/−^ Rpl22^wt/HA^ offspring, the floxed exon 4 is deleted and the tagged exon 4 is expressed selectively in myeloid cells. 8 weeks after tamoxifen injection, only long-lived myeloid cells including microglia carry HA-tagged ribosomes. All experiments randomly included both male and female mice aged between 8 and 10 weeks. Mice were housed in ventilated cages on 12-hour light: dark cycles under specific pathogen-free condition. All animal experiments were approved by local administration in Germany and were performed in strict conformity to respective national, federal and institutional regulations and the guidelines of the Federation of European Laboratory Animal Science Association (Identification number: 22/0055 and 18/2899).

### Preparation of microglial cultures

Microglia were generated from cortices of day 3 post-natum newborn pups as previously described [72]. Briefly, cortices were extracted in ice-cold Hanks Balanced Salt Solution (Gibco, 14065-056) containing 4-(2-hydroxyethyl)-1-piperazineethanesulfonic acid (HEPES; Gibco, 15630-056) and the meninges were removed under the stereomicroscope. The cortical tissue was finely minced and digested with 0.1% trypsin (Capricorn, TRY-1B10) for 10 min at 37 °C on a shaker and 0.25% DNase (Roche, 11284932001) was added. Mixed glial culture progenitor (MGP+; Capricorn, DMEM-HXA) medium supplemented with 10% fetal bovine serum (Capricorn, FBS-11 A) and 1% penicillin/streptomycin (Capricorn, PS-B) were added. The homogenate was centrifuged, and the supernatant was removed. The pellet was re-suspended in triturating solution, triturated several times with 100 µm strainer and seeded into poly-L-lysine pre-coated flasks with MGP + medium at 37 °C and 5% CO_2_. The medium was changed at day 1 and 5. After 10 days of mixed glial culture, microglia were harvested by shaking the flasks (125 rpm) for 40 min at 37 °C. Microglial cells were re-plated at a density of 2.0 × 10^5^ cells per well in 24 well plates and incubated at 37 °C and 5% CO_2_. Microglia were used the following day for experiments.

### Tamoxifen administration

To induce expression of HA epitopes in microglial ribosomes, CX3CR1-Cre^ERT2+/−^ Rpl22^HA/wt^ mice were subcutaneously injected with 4 mg of tamoxifen (Sigma, T5648) suspended in 200 µL of corn oil (Sigma, C8267) for two consecutive days. All animals were tamoxifen-treated first at age of 6 weeks. Mice were ready for experiment 8 weeks after initial tamoxifen injection, thus allowing complete turnover of CX3CR1^+^ circulating cells.

### VSV infection

For in vivo VSV infection, mice aged 14 weeks were intranasally instilled with 10 µL of 10^3^ PFU VSV-Indiana (Mudd-Summers isolate). Control animals received the same volume of vehicle solution (PBS). Six days post infection, the animals were sacrificed by cervical dislocation prior to brain harvesting.

For in vitro VSV treatment, culture microglia were inoculated at MOI of 0.5 with VSV diluted in DMEM medium for 1 h at 37 °C in 5% CO_2_. At 8 h post infection, supernatant was removed, and the adherent cells were lysed by TRIzol (ThermoFischer, 15596018), harvested and stored at -80 °C until further use.

### Tissue immunoprecipitation

Immunoprecipitation of ribosomes in CX3CR1-Cre^ERT2+/−^ Rpl22^HA/wt^ mice was performed as previously described, though with minor modifications [17]. In brief, the brain tissue was placed in a prechilled 2 mL Dounce homogenizer and 500 µL lysing buffer was added containing 50 mM Tris-HCl (pH 7.4), 12 mM MgCl_2_ (ThermoFischer, AM9530G), 100 mM KCl (ThermoFischer, AM9640G), 1 mM DTT (Sigma, D9779), 200 U/mL RNasin (Promoga, N2515), 1% NP-40 (Sigma, CA-630), 1× protease inhibitor cocktail (Merck, 4693159001), 100 µg/mL cycloheximide (Sigma, C7698) and 1 mg/mL heparin (Sigma, H3149). Samples were centrifuged at 4 °C at 10,000xg for 10 min, and supernatant was collected. 50 µL of the lysate was aliquoted and was kept as “input fraction”. 10% v/v of re-suspended HA-specific antibody beads (ThermoFischer, 88836) was added to the remaining supernatant, and RiboTag-IP fractions were rotated at 4 °C overnight. Afterwards, samples were washed 3 times with high salt-buffer (50 mM Tris-HCl (pH 7.4), 300 mM KCl, 12 mM MgCl_2_, 100 µg/mL Cycloheximide, 1 mM DTT and 1% NP-40), 5 min per wash in the cold room on end-to-end rotator. After washes, ribosome-RNA bound complexes were magnetized and excess buffer removed. The beads were re-suspended in 350 µL RLT buffer supplemented with 1% β-mercaptoethanol (Sigma, M6250).

### RNA isolation

RNA from “input” and RiboTag-IP fractions was extracted using Qiagen RNeasy Mini Kit following manufactures instruction. For in vitro cultured microglia, RNA was isolated using Direct-zol RNA Miniprep Plus Kit (Zymo Research). RNA quality and concentrations were accessed using Bioanalyzer RNA kit (Agilent) and samples with RIN value > 8.0 were selected for further processing.

### Bulk RNA sequencing and analysis

Library preparation of selected samples was performed with NEBNext Low input RNA Library Prep Kit for Illumina (NEB, E6420) following manufacturer’s instructions. Constructed cDNA libraries were sequenced on an Illumina NovaSeq-6000 platform with a 50 bp paired-end read configuration. Quality of raw fastq-files was assessed using FastQC software (version 0.11.9) and mapped to reference genome assembly of *Mus musculus* (GRCm38) from Ensembl using STAR v2.5.4b software [73]. Only reads with unique mapping were considered for downstream analysis. Gene-level read counts were obtained with FeatureCounts [74]. Prior to differential analysis, batch effect adjustment was performed using ComBat-seq [22]. Differential expression analysis was performed using DESeq2 package [75] in R environment (version 1.26.0). We used both Wald test of the negative binomial model coefficients (DESeq2-Wald) as well as likelihood ratio test compared with reduced model (DESeq2-LRT) to test significance of gene expression differences as a function of samples at an absolute log2-fold change threshold of 1. To control false discovery rate (FDR), test p-values were adjusted to multiple comparisons using the Benjamini-Hochberg procedure. To display expression levels of selected gene signatures between samples, raw counts were normalized based on median of ratios method in DESeq2. In brief, DESeq2 generates normalized reads by dividing counts with sample-specific size factors that were determined by the median ratio of gene counts relative to the geometric mean per gene [76]. We exploited this approach to enable accurate comparisons of gene expression levels between different samples, ensuring that the differences observed reflect true biological variability rather than technical artifacts. Visualizations of differentially expressed genes represented as heatmaps were generated using standardized functions of normalized counts from DESeq2 analysis. Optimal k-means clusters were obtained using elbow method.

### Exploration of the underlying biological characteristics

Functional annotation of differentially expressed gene signatures was performed in Gene Ontology (GO), subcategory “Biological process” using clusterProfiler Bioconductor package [77]. Specifically, over representation analysis (ORA) implemented in the package was used to determine enrichment of GO terms with FDR of *q* < 0.05. The *p* values were calculated using hypergeometric test and adjusted for multiple comparison with the Benjamini–Hochberg method. Gene set enrichment analysis (GSEA) [78] was performed to determine whether a pre-defined set of DEGs that associated with GO terms shows statistical significance by ranking normalized enrichment score (NES). GO term was considered enriched with a NES > 1 and was predicted de-enriched with NES <-1.

### Processing and analysis of archived datasets

We assembled a compendium of seven published scRNA-seq studies and one-bulk RNA-seq study. For scRNA-seq, raw count matrices or loom files containing count matrices were obtained from Gene Expression Omnibus (GEO) database or laboratory websites. Datasets that composed of different genotypes, treatments and gender, only samples from WT, controls, or mixed gender mice were selected for downstream analysis.

Von Hove et al. [23], full aggregated raw gene-cell count matrix consisting of dura, choroid plexus, enriched subdural meninges, macrophages and whole brain of WT mice were obtained from GSE128855. The study used 10X genomics sample preparation protocol, antibody-based cell sorting to cells and sequenced with 10X genomics platform. Metadata, including cell type annotations, were retrieved from Brain Immune Atlas.

Zhong et al. [29], processed digital gene expression matrix from the whole bone marrow of healthy WT mice were downloaded from GEO (GSE182986). Femur and tibia were dissociated with enzymatic digestion and libraries of resulting cell suspensions were constructed with the Singleron protocol. Sample and cell-level metadata, including cell type annotations, were retrieved from the authors.

Kimmel et al. [30], sparse matrices from the spleen of 4 adult WT mice (7–8 months) were obtained from GEO (GSE132901). Spleen was mechanically dissociated and each tissue cell suspension from each animal was processed in two technical replicates using the 10X protocol. Metadata and cell type annotations were retrieved from the original publication.

Tepe et al. [26], raw digital gene expression matrix from the olfactory bulb of adult WT mice were downloaded from GSE121891. Tissues were dissociated according to 10X genomics sample preparation protocol. Metadata was obtained from the same source.

Hammond et al. [31], processed digital gene expression matrices from whole brain of 2 male and 2 female adult WT mice (P100) were retrieved from GEO (GSE121654). Tissues were mechanically dissociated under ice cold conditions, cells enriched via FACS and sequenced using 10X genomics. Metadata, including cell type annotations, were obtained from the supporting information files accompanying the publication.

Marsh et al. [27], raw count matrices from whole brain of 4 adult mice (P89-P90) were downloaded from GSE152183. The study used modified 10X genomic sample preparation protocol with inclusion of transcriptional inhibitors. Metadata, including biological replicates, experimental conditions and cell type annotations, were obtained from the supporting information files accompanying the published manuscript.

Rosen et al. [33], raw sparse count matrix from murine cortical and hippocampal tissues were downloaded from GSE212199. Tissues were dissociated using Miltenyi dissociation protocol and the resulting cell suspensions were processed with Chromium Single Cell 3′ Library Kit. Meta files including experimental conditions and biological replicates were obtained from the same source.

For bulk RNA-seq from Chhatbar et al. [4], raw paired-end fastq-files from CX3CR1-Cre^ERT2+/−^TdTomato^wt/ST^ adult mice (P100) challenged with VSV and matched controls were obtained from GEO (GSE110188). Brain tissues were processed using Miltenyi dissociation protocol and pre-sorted td-tomato^+^ cells via FACS. Metadata including biological replicates and experimental conditions were aggregated from the publication. Data processing and differential expression analysis was performed with aforementioned bulk RNA-seq analysis pipeline.

Seurat version 4 was used for the single-cell analysis [79]. For scRNA-seq datasets, an initial round of cleaning and clustering was performed using dataset-specific parameters described in the original publications. Cluster identities were determined by calculating enriched markers using the FindAllMarkers () function implement in Seurat. Cell types were assigned by identifying genes that were unique to each cluster and through cross-referencing to known markers of each cell type and existing published datasets.

To study microglia, we applied a uniform processing pipeline to process each dataset. Microglia clusters were identified in each dataset with canonical markers and selected for downstream analysis. A second round of microglial clustering was performed on each dataset separately using defined set parameters. In brief, SCTranform () function was used to normalize and scale data, then UMIs and mitochondrial gene percentages per cell were regressed out. Principal component analysis (PCA) was performed with the first 30 PCs, and a by shared nearest neighbor (SNN) graph was build using 10 dimensions. Clusters were identified using the Louvian-clustering algorithm at a resolution of 0.8, and a graph of all cell populations was generated through uniform manifold approximation and projection (UMAP) using 10 PCs.

### Data correction and integration

Prior to integrative analysis, we first performed normalization of pre-selected microglia cell clusters from Van Hove et al. [23], Tepe et al. [26], Hammond et al. [31], Marsh et al. [27] datasets with Seurat. All datasets were merged, and low-quality cells were filtered out based on threshold cut-off for minimum numbers of genes detected at 200 genes per cell and maximum mitochondrial percentage of 10. Gene expression of split datasets was normalized and transformed using SCTransform () function to identify highly variable genes (HVG). The top 3000 HVG were used as input to identify cross-dataset pairs of cells that are in matched biological state (anchors) to integrate the datasets together. Due to differences in cell isolation and handling protocols, library preparation technology, and sequencing platforms across the datasets, we then performed iterative linear correction based on soft clustering implemented in Harmony [80] to integrate technical covariates in a low-dimensional space. The output was used for linear dimensionality reduction using 11 PCs, followed by application of the non-linear UMAP dimensionality reduction with resolution of 0.8. UMAP plots and gene expression plots were generated using built-in Seurat/ggplot2 plotting functions.

### Differential gene expression analysis

The significance of inter-dataset gene expression of microglia was determined using the MAST package [34]. In brief, the integrated Seurat object was log transformed and converted into SCA object. Genes expressed in less than 10% of cells were filtered out and identity of each dataset (batch) was assigned as predictor. We fitted a hurdle model, modelling the batch and centered ‘ngeneson’ factor to adjust for the cellular detection rate. Likelihood ratio test (LRT) was performed to test the differences across the datasets. Only genes that attained cutoff criteria of FDR < 0.05 and log2-fold change >|0.25| were selected for downstream analysis.

### Gene signature scoring

To define cell type enrichment of cell activation signature identified from bulk RNA-seq analysis, the UCell package was used [25]. UCell is a gene signature scoring method based on Mann-Whitney U statistics. In brief, the algorithm calculates the relative gene expression in individual cells and ranks genes for each cell for each gene set to generate UCell scores. In the end, UCell scores depend only on the relative expression of genes in each cell and are not dependent on dataset composition. UCell enrichment scores were projected on UMAP using FeaturePlot function implemented in Seurat.

### In silico deconvolution

For deconvolution analysis, we utilized scRNA-seq datasets of microglia from Rosen et al. [33] as reference. We evaluated cell-type proportions in IP microglia using Single-cell RNA Quantity Informed Deconvolution (SQUID) [36], which combines bulk RNA-seq transformation and dampened weighted least squares deconvolution approaches to predict the composition of cell mixtures.

### Immunohistochemistry

Mice were anesthetized with a ketamine/xylazine and transcardially perfused with ice cold 1x PBS followed by ice cold 4% PFA. Brains were harvested, post-fixed in 4% PFA for 4 h and dehydrated in 30% sucrose overnight at 4 °C. Brains were then embedded in Tissue-Tek optimal cutting temperature compound (Sakura, 4583) and cut into 7 μm sagittal sections. Prior to immunolabeling, slices were rehydrated with 0.5% Triton-X100 in PBS for 15 min. Tissue slices were blocked in tissue blocking solution (PBS containing 5% donkey serum [Sigma, S30M] and permeabilized with 0.5% Triton-X 100 [BioRad, 1610407]) for 1 h at room temperature (RT). Primary antibodies: CD3 (1:100; Biolegend, 100202) and IBA1 (1:500; Abcam, ab5076) were added and incubated overnight at 4 °C. Excess antibodies were removed through 3 washing steps with 0.5% Triton-X100 in PBS for 5 min followed by secondary antibodies labeling with donkey anti-rat AF568 (1:500; Abcam, 175475) and donkey anti-goat AF647 (1:500; Invitrogen, A21447) for 1 h at RT. Following secondary staining, sections were washed 3 times with 0.5% Triton-X100 in PBS. Nuclei were stained with DAPI (1:1000; Sigma D9542) for 2 min, dried and coverslips were mounted using Dako fluorescence mounting medium. Fluorescent images were acquired with Olympus FV3000 and analyzed with ImageJ.

### Cell sorting and flow cytometry

Mice were euthanized and perfused with ice cold 1x PBS. Brains were isolated and dissociated by enzymatic digestion in a 2 mL RPMI (Capricorn, RMPI-XA) containing 20% collagenase (Sigma, C9263), 40% DNase and 5% FBS in gentleMACS C tubes 3 times for 8 min at 37^°^C. The homogenates were loaded on a three layered Percoll gradient (30, 37 and 70%; Sigma, P1644) and centrifuged at 500 g for 30 min. Immune cells were collected at 37% and 70% Percoll interphase, washed in PBS and immediately processed for downstream analysis. For sorting experiments, freshly isolated cells were incubated with anti-FcγIII/II receptor antibody (CD16; Biolegend, 156603) for 10 min and thereafter immunolabeled for CD45.2 PacBlue (Biolegend, 109820). An aliquot of 250 µl was kept aside as “unsorted” fraction. After cell sorting, both the “sorted” and “unsorted” fractions were immunolabeled with the following flourochrome-conjugated antibodies: CD11b APC-Cy7 (Biolegend, 101226), P2RY12 APC (Biolegend, 848006), CD74 AF647 (Biolegend, 151004), LY6C AF700 (Biolegend, 128024), CX3CR1 BV510 (Biolegend, 149025), MHCI FITC (Biolegend, 125508), LY6G PE-Cy7 (Biolegend, 127618) and MHCII PE-Cy5 (Biolegend, 107611). Samples were incubated for 20 min at 4^°^C, washed and re-suspended in 300 µL of FACS buffer.

For flow cytometric analysis of brain immune cells during VSV infection, the freshly isolated cells were incubated with anti-FcγIII/II receptor antibody (CD16; Biolegend, 156603) to prevent unspecific binding of the antibodies. Thereafter, cells were further labeled with the following flourochrome-conjugated antibodies against cell surface markers in FACS buffer: CD45.2 PacBlue (Biolegend, 109820), CD11b APC-Cy7 (Biolegend, 101226), LY6C AF700 (Biolegend, 128024), LY6G PE-Cy7 (Biolegend, 127618), CX3CR1 BV510 (Biolegend, 149025), MHCI BUV661 (BD, 749702), MHCII BV785 (Biolegend, 107645), CD4 BV570 (Biolegend, 100541), CD8b BV711 (Biolegend, 126633), CD11c BV605 (BD, 563057), TCR-β PE-dazzle (Biolegend, 109240), B220 PE-Cy5 (Invitrogen, RM2618) and NK1.1 BV421 (Biolegend, 108732). Samples were incubated for 20 min at 4 °C, washed and re-suspended in 300 µL of FACS buffer. All samples were acquired on a Sony ID7000 flow cytometer equipped with 405, 488, 561 and 633 lasers. Obtained data were analyzed with FlowJo software (Tree Star).

### Dimensional reduction and clustering analysis of flow cytometry data

Unsupervised clustering of flow cytometry data was performed with FlowSOM in R environment. First, non-lymphocyte cells and doublets were excluded based on FSC/SSC parameters. Then, fcs files containing both CD45^+^CD11b^+^ and CD45^+^CD11b^−^ cells from each sample were exported for further analysis. Marker intensities were transformed with biexponentialTransform () function, scaled and reduced into low-dimensional space using BuildSOM () function in FlowSOM algorithm. FlowSOM clusters cells with similar phenotypic appearance into subpopulation or subsets, which are depicted in special proximity on the UMAP plot. Optimal clusters were iteratively estimated based on delta area generated by ConsensusClusterPlus.

### Statistics and reproducibility

Statistical analysis and data visualization in the present study was performed by using the R software (version 4.1.1, http://www.r-project.org). Unless specifically stated, p or FDR values < 0.05 were considered statistically significant.

### Electronic supplementary material

Below is the link to the electronic supplementary material.

**Supplemental Fig. 1**: Expression of cell activation signatures in bulk RNA and scRNA-seq. A, Box plot with overlaid dotplot of normalized counts revealing prominent expression of aberrant activation signatures in sorted microglia (for additional details see caption of Fig. 1). **B**, Violin plots of basic scRNA-seq quality metrics and UMAP embedding of 21,463 cells from Van Hove *at al.* [23], highlighting major cell types. **C**, Heatmap depicting the scaled and log-normalized expression values of the top three most highly enriched genes in microglia clusters. A maximum of 100 cells per cluster are displayed. **D**, Cell-specific expression of selected cell activation genes overlaid on the UMAP coordinates. Color scale represents gene-weighted kernel density estimates.

**Supplemental Fig. 2**: Integrative analysis of publicly archived large-scale transcriptomic datasets. Basic quality metrics and UMAP embedding of scRNA-seq datasets of brain immune cells from **(A)** Hammond et al. [31], **(B)** Marsh et al. [27], and **(C)** Tepe et al. [26]. Violin plot displaying distribution of cell activation enrichment score in cell types from **(D)** bone marrow and **(E)** splenocytes. OPC, oligodendrocyte progenitor cells; OEC, olfactory epithelial cells; RBC, red blood cells; DC, dendritic cells; HSC, hematopoietic stem cells; MSC, mesenchymal stem cells; NMP, neutrophil-myeloid progenitors.

**Supplemental Fig. 3**: Expression of cell activation signatures in integrated scRNA-seq with key experimental variations. A, UMAP plot of 25,406 integrated microglia cells depicting three microglial clusters. Violin plot displaying distribution of cell activation enrichment score in integrated microglia grouped by **(B)** dataset and **(C)** microglia subtypes. **D**, Abundance of selected key cell activation genes in the integrated microglia datasets. **E**, Average expression of canonical microglial markers across the integrated datasets. **F**, Violin plot showing expression of key cell activation genes in microglia treated with transcriptional inhibitors. **G**, Contour plots representing the gating strategy for myeloid cells. Gates are indicated in blue. Doublets and dead cells were excluded before. Both CD45^+^CD11b^+^ and CD45^+^CD11b^−^ populations were exported for dimensionality reduction and clustering, whereas CD45^+^CD11b^+^ were further gated for the microglia population. **H**, Contour plots representing CD74^+^, MHCI^+^ or MHCII^+^ expressing microglia.

**Supplemental Fig. 4**: Viral infection of the brain induces transcriptional shift of microglia. A, Venn diagram showing intersection of differentially regulated genes between VSV versus PBS in IP, in vitro and sorted samples. **B**, Roseplot showing directional distributions of overlapping genes regulated in IP, in vitro, and sorted microglia based on expression changes. **C**, Volcano plot highlighting fold change of differentially regulated genes between VSV versus PBS from IP microglia. Genes with up- or downregulated expression are highlighted in amber and pink, respectively. **D**, UMAP plot of scRNA-seq datasets from Rosen et al. [33] depicting segregation of immune cells based on treatment. Microglia isolated from controls segregated from microglia isolated from WNV-infected mice. **E**, UMAP plot displaying density expression levels of genes encoding for MHC complexes and phagocytosis in *Cx3cr1* expressing microglia. **F**, UMAP depicting immune composition in the brain following VSV infection (left) and surface maker expression associated with identified clusters (right).

**Supplemental table 1**: Cell activation gene signatures of microglia and consensus gene list for the gene scoring module.

## Data Availability

The RNA-seq data generated in this study have been deposited in the NCBI Gene Expression Omnibus and are accessible through GEO Series accession number GSE271293. No original codes were generated in this study. Further information and requests for resources and reagents should be directed to the lead contact.
