# Supplementary figures and images for: Transcriptomic analysis unveils bona fide molecular signatures of microglia under conditions of homeostasis and viral encephalitis
Source: J Neuroinflammation. 2024 Aug 17;21:203. doi: 10.1186/s12974-024-03197-2 (PMC11330067; doi:10.1186/s12974-024-03197-2)

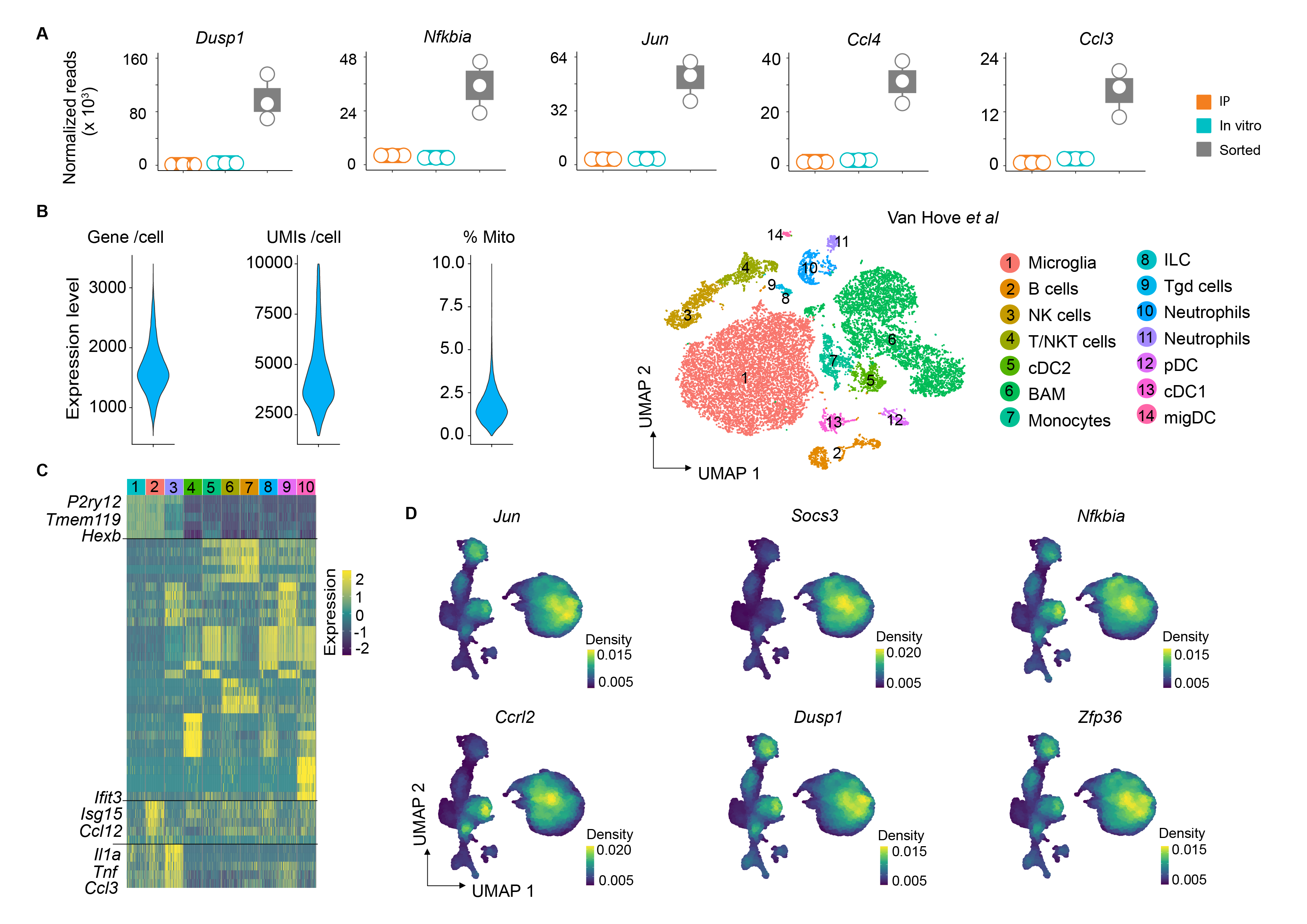

Supplement: Supplementary file 1 — Supplemental Fig. 1: Expression of cell activation signatures in bulk RNA and scRNA-seq. A, Box plot with overlaid dotplot of normalized counts revealing prominent expression of aberrant activation signatures in sorted microglia (for additional details see caption of Fig. 1). B, Violin plots of basic scRNA-seq quality metrics and UMAP embedding of 21,463 cells from Van Hove at al. [23], highlighting major cell types. C, Heatmap depicting the scaled and log-normalized expression values of the top three most highly enriched genes in microglia clusters. A maximum of 100 cells per cluster are displayed. D, Cell-specific expression of selected cell activation genes overlaid on the UMAP coordinates. Color scale represents gene-weighted kernel density estimates. [file 12974_2024_3197_MOESM1_ESM.tif]

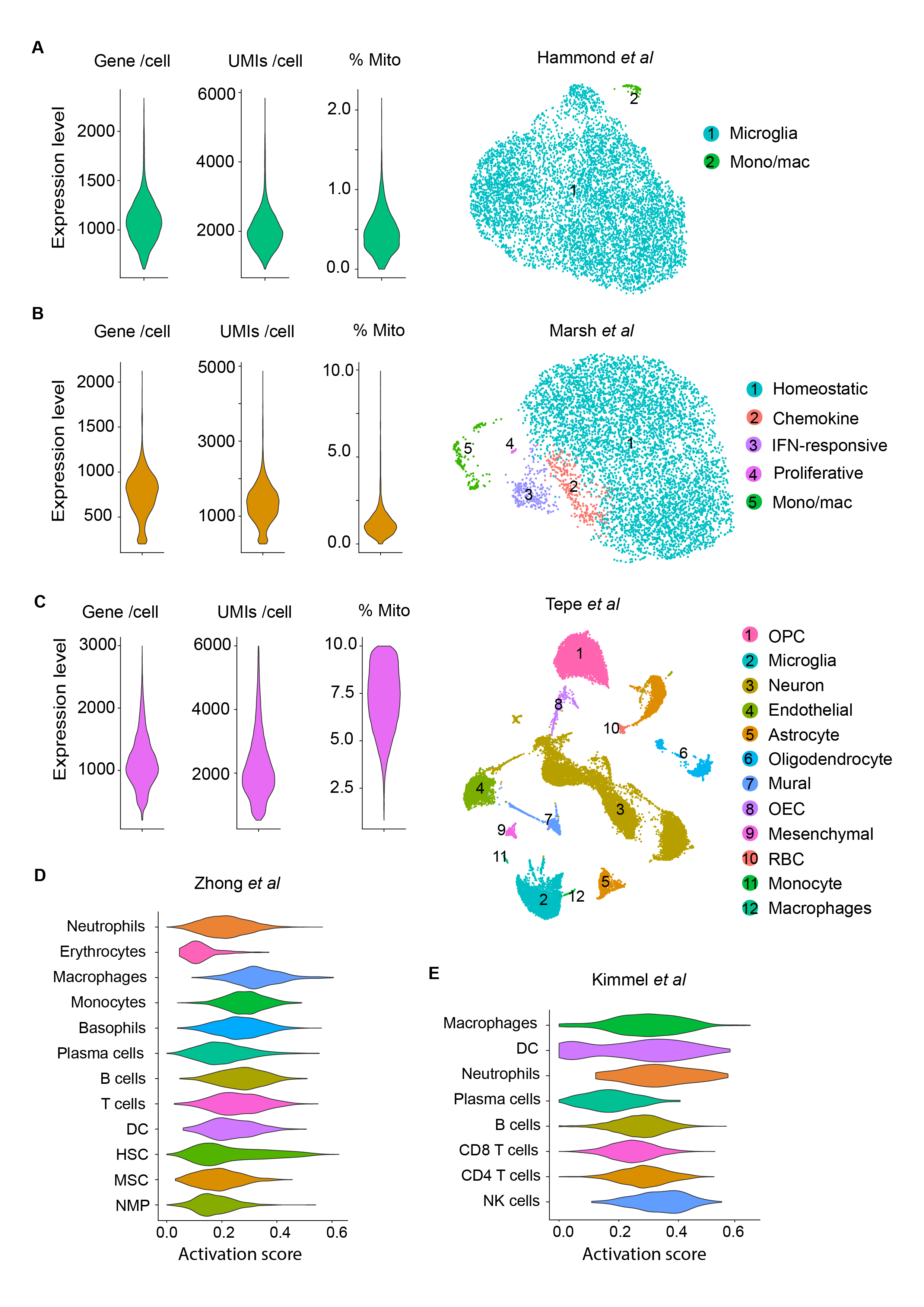

Supplement: Supplementary file 2 — Supplemental Fig. 2: Integrative analysis of publicly archived large-scale transcriptomic datasets. Basic quality metrics and UMAP embedding of scRNA-seq datasets of brain immune cells from (A) Hammond et al. [31], (B) Marsh et al. [27], and (C) Tepe et al. [26]. Violin plot displaying distribution of cell activation enrichment score in cell types from (D) bone marrow and (E) splenocytes. OPC, oligodendrocyte progenitor cells; OEC, olfactory epithelial cells; RBC, red blood cells; DC, dendritic cells; HSC, hematopoietic stem cells; MSC, mesenchymal stem cells; NMP, neutrophil-myeloid progenitors. [file 12974_2024_3197_MOESM2_ESM.tif]

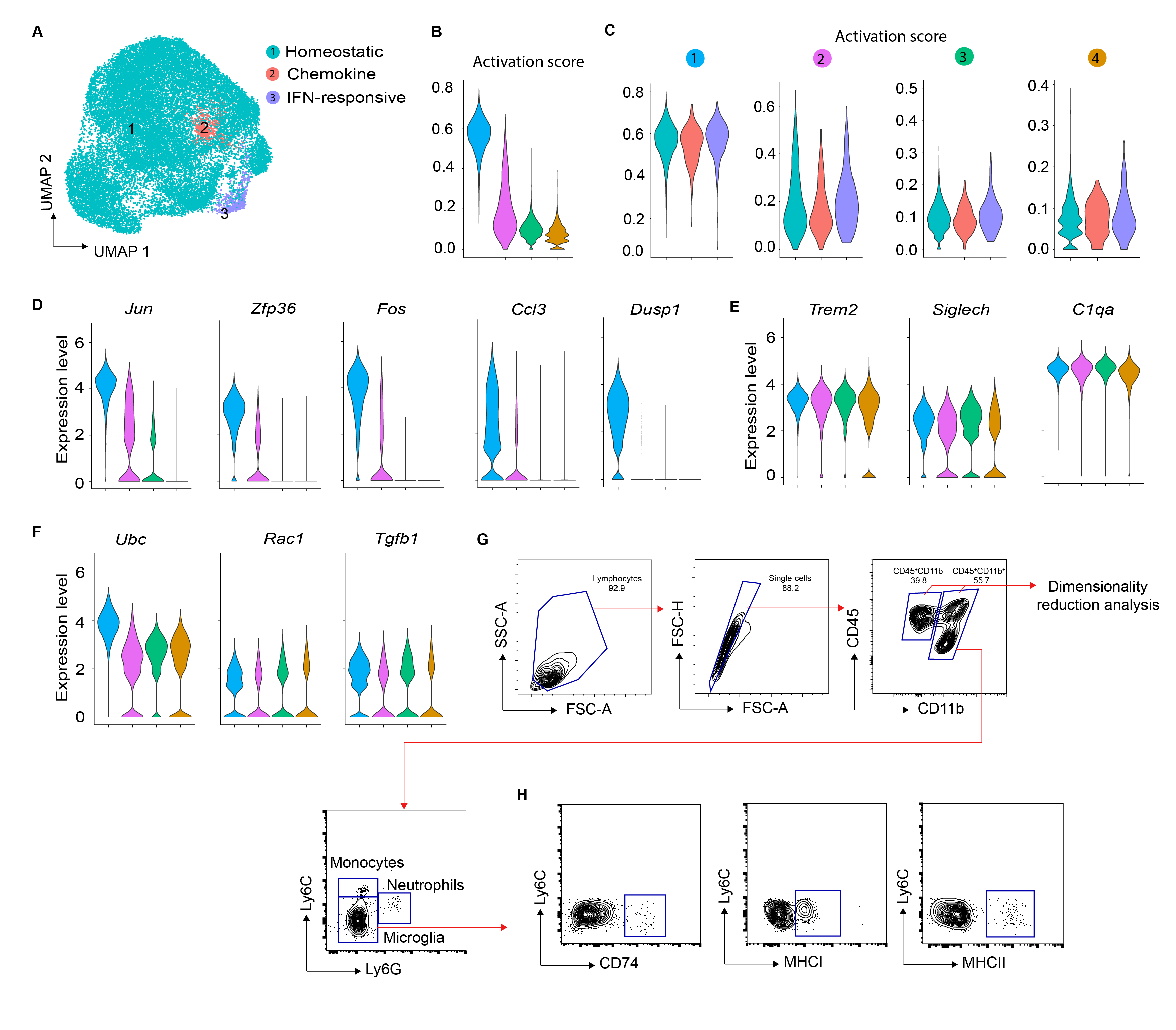

Supplement: Supplementary file 3 — Supplemental Fig. 3: Expression of cell activation signatures in integrated scRNA-seq with key experimental variations. A, UMAP plot of 25,406 integrated microglia cells depicting three microglial clusters. Violin plot displaying distribution of cell activation enrichment score in integrated microglia grouped by (B) dataset and (C) microglia subtypes. D, Abundance of selected key cell activation genes in the integrated microglia datasets. E, Average expression of canonical microglial markers across the integrated datasets. F, Violin plot showing expression of key cell activation genes in microglia treated with transcriptional inhibitors. G, Contour plots representing the gating strategy for myeloid cells. Gates are indicated in blue. Doublets and dead cells were excluded before. Both CD45+CD11b+ and CD45+CD11b− populations were exported for dimensionality reduction and clustering, whereas CD45+CD11b+ were further gated for the microglia population. H, Contour plots representing CD74+, MHCI+ or MHCII+ expressing microglia. [file 12974_2024_3197_MOESM3_ESM.tif]

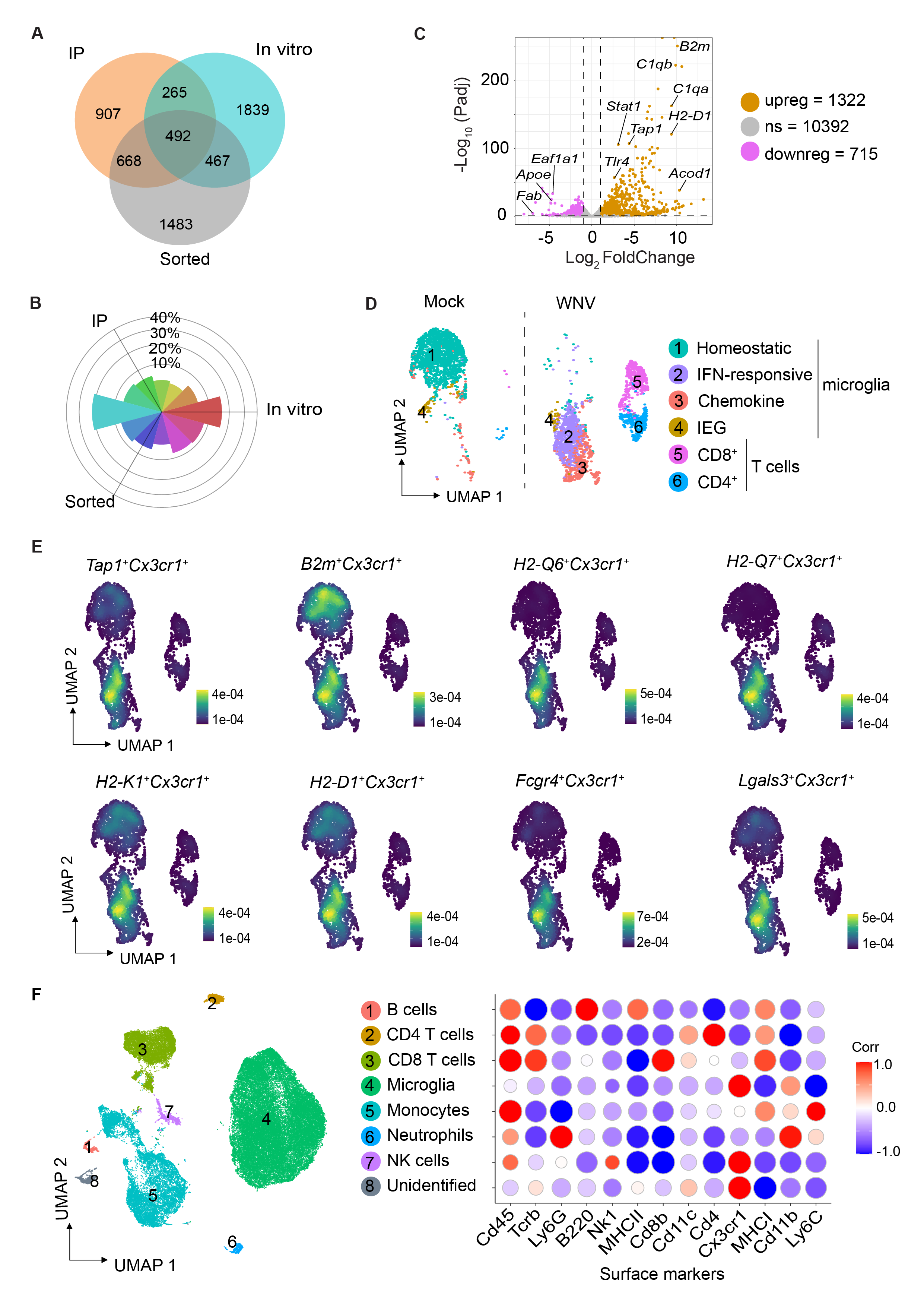

Supplement: Supplementary file 4 — Supplemental Fig. 4: Viral infection of the brain induces transcriptional shift of microglia. A, Venn diagram showing intersection of differentially regulated genes between VSV versus PBS in IP, in vitro and sorted samples. B, Roseplot showing directional distributions of overlapping genes regulated in IP, in vitro, and sorted microglia based on expression changes. C, Volcano plot highlighting fold change of differentially regulated genes between VSV versus PBS from IP microglia. Genes with up- or downregulated expression are highlighted in amber and pink, respectively. D, UMAP plot of scRNA-seq datasets from Rosen et al. [33] depicting segregation of immune cells based on treatment. Microglia isolated from controls segregated from microglia isolated from WNV-infected mice. E, UMAP plot displaying density expression levels of genes encoding for MHC complexes and phagocytosis in Cx3cr1 expressing microglia. F, UMAP depicting immune composition in the brain following VSV infection (left) and surface maker expression associated with identified clusters (right). [file 12974_2024_3197_MOESM4_ESM.tif]
